# A hierarchical Bayesian network approach for linkage disequilibrium modeling and data-dimensionality reduction prior to genome-wide association studies

**DOI:** 10.1186/1471-2105-12-16

**Published:** 2011-01-12

**Authors:** Raphaël Mourad, Christine Sinoquet, Philippe Leray

**Affiliations:** 1 LINA, UMR CNRS 6241, Ecole Polytechnique de l'Université de Nantes, rue Christian Pauc, BP 50609, 44306 Nantes Cedex 3, France; 2 LINA, UMR CNRS 6241, Université de Nantes, 2 rue de la Houssinie're, BP 92208, 44322 Nantes Cedex, France

## Abstract

**Background:**

Discovering the genetic basis of common genetic diseases in the human genome represents a public health issue. However, the dimensionality of the genetic data (up to 1 million genetic markers) and its complexity make the statistical analysis a challenging task.

**Results:**

We present an accurate modeling of dependences between genetic markers, based on a forest of hierarchical latent class models which is a particular class of probabilistic graphical models. This model offers an adapted framework to deal with the fuzzy nature of linkage disequilibrium blocks. In addition, the data dimensionality can be reduced through the latent variables of the model which synthesize the information borne by genetic markers. In order to tackle the learning of both forest structure and probability distributions, a generic algorithm has been proposed. A first implementation of our algorithm has been shown to be tractable on benchmarks describing 10^5 ^variables for 2000 individuals.

**Conclusions:**

The forest of hierarchical latent class models offers several advantages for genome-wide association studies: accurate modeling of linkage disequilibrium, flexible data dimensionality reduction and biological meaning borne by latent variables.

## Background

Genetic markers such as SNPs are the key to dissecting the genetic susceptibility of common complex diseases, such as asthma, diabetes, atherosclerosis and some cancers [1]. The purpose is identifying combinations of genetic determinants which should accumulate among affected subjects. Generally, in such combinations, each genetic variant only exerts a modest impact on the observed phenotype, whereas, in contrast, the interaction between genetic variants and, possibly, environmental factors is determinant. Decreasing genotyping costs now enable the generation of hundreds of thousands of SNPs, spanning the whole human genome, across cohorts of cases and controls. This scaling up to genome-wide association studies (GWASs) makes the analysis of high-dimensional data a hot topic [2]. Despite recent technological advances and extensive research effort, the genetic basis of the aforementioned diseases remains to a large extent unknown. Yet, the search for associations between single SNPs and the variable describing case/control status requires carrying out a large number of statistical tests. Since SNP patterns, rather than single SNPs, are likely to be determinant for complex diseases, a high rate of false positives as well as a perceptible statistical power decrease, not to mention intractability, are severe issues to be overcome.

The simplest type of genetic polymorphism, single nucleotide polymorphism (SNP), involves only one nucleotide change, which occurred generations ago within the DNA sequence. To fix ideas, we emphasize that one single individual can be uniquely defined by only 30 to 80 independent SNPs and unrelated individuals differ in about 0.1% of their 3.1 billion nucleotides [3]. Compared with other kinds of DNA markers, SNPs are appealing because they are abundant, genetically stable and amenable to high-throughput automated analysis. Consistently, advances in high-throughput SNP genotyping technologies lead the way to various down-stream analyses, including GWASs.

Exploiting the existence of statistical dependences between neighboring SNPs, also called linkage disequilibrium (LD), is the key to association study achievement [4]. Indeed, a causal variant (*i.e*. a genetic factor) may not be a SNP. For instance, insertions, deletions, inversions and copy-number polymorphisms may be causative of disease susceptibility. Nevertheless, a well-designed study will have a good chance of including one or more SNPs that are in strong LD with a common causal variant. In the latter case, indirect association with the phenotype, say affected/unaffected status, will be revealed (see Additional file 1).

Interestingly, LD also offers solutions to reduce data dimensionality in GWASs. In the human genome, LD is highly structured into the so-called "haplotype block structure" [5]: regions where statistical dependences between contiguous markers (called blocks) are high alternate with shorter regions characterized by low statistical dependences (see Additional file 2). The most likely explanation of this phenomenon is related to the presence of large regions with low recombination rates separated by recombination hotspots (*i.e*. small specific regions with high recombination rates) [6]. Relying on this feature, various approaches were proposed to achieve data dimensionality reduction: testing association with haplotypes (*i.e*. inferred data underlying genotypic data) [7], partitioning the genome according to spatial correlation [8], selecting SNPs informative about their context, or SNP tags [9] (for more references, see [10] for example). Recent methods, such as HaploBuild [11], have permitted to construct more biologically relevant haplotypes where the "haplotype cluster structure", instead of the "haplotype block structure", is assumed: haplotypes are not constrained by contiguous orientation. Unfortunately, these methods do not take into account all existing dependences since they miss higher-order dependences. Actually, these methods do not consider the fuzzy nature of LD: the LD block boundaries are not accurately defined over the genome (see Additional file 3).

Due to their ability to represent conditional independences between variables, probabilistic graphical models (PGMs) offer an adapted framework for an accurate modeling of dependences between SNPs. A PGM is a probabilistic model relying on a graph representing conditional independences within a set of random variables. Inherently, this model simplifies the description of the joint distribution of the set of variables. Several subclasses of PGMs exist such as Markov random fields (MRFs) and Bayesian networks (BNs). The main difference between these two subclasses remains in the nature of the graph: in contrast with MRFs, Bayesian networks are directed graphs. Although the observed variables (OVs) are often sufficient to describe their joint distribution, sometimes, additional unobserved variables, also named latent variables (LVs), have a role to play.

Only few research works have been dedicated to SNP dependence modeling through PGMs. A hard task because of high data dimensionality, tackling this modeling issue through PGMs nevertheless offers an attractive lead. Approaches based both on MRFs [12] and BNs have been designed. Regarding the latter, some methods only consider observed variables [13,14] whereas other models include latent variables [15,16]. In particular, hierarchical BNs are the most promising models for LD representation: their hierarchical structure supported by LVs allows flexible information synthesis, thus efficiently reducing the data dimensionality. To our knowledge, modeling LD through hierarchical BNs in order to reduce SNP data dimensionality has not yet been designed. Notably, scalability remains a crucial issue for GWASs.

In this paper, we emphasize the interest of using a forest of hierarchical latent class models (FHLCMs), to reduce the dimension of the data to be further submitted to statistical analyses devoted to the discovery of genetic factors potentially involved in the disease. Such studies encompass single-SNP analysis [17], multiple-SNP analysis [18], SNP-SNP interaction analysis [19] and analysis integrating gene expression [20,21]. An FHLCM is a hierarchical BN with discrete observed and latent variables. Basically, latent variables capture the information borne by underlying markers. In their turn, latent variables are clustered into groups and, if relevant, such groups are subsequently subsumed by additional latent variables. Iterating this process yields a hierarchical structure. First, the great advantage to GWASs is that further statistical analyses can be chiefly performed on latent variables. Thus, a reduced number of variables will be examined. Second, a model based on a hierarchical structure provides a flexible data mining tool. For example, different degrees of data dimensionality reduction are available to the statistician. Moreover, the hierarchical structure is meant to efficiently conduct refined association testing: zooming in through narrower and narrower regions in search for a stronger association with the disease ends pointing out the potential markers of interest.

However, most algorithms dedicated to the learning of hierarchical latent class models (HLCMs) fail the scalability criterion when the data describe thousands of variables and a few hundreds of individuals. In a previous work-on progress paper [22], we designed an algorithm devoted to learning FHLCMs. This algorithm was named CFHLC, which stands for Construction of Forests of Hierarchical Latent Class models. The contribution brought in the present extended version is the following: (i) we advocate the use of FHLCMs to model LD; (ii) we provide a detailed description of the main concepts underlying our approach; (iii) using real data, we show that the FHLCM graph is representative of the haplotype cluster structure; (iv) in addition, we compare the haplotype cluster structure obtained through CFHLC with those output by four other algorithms; (v) relying on both real and simulated data, we demonstrate the ability of FHLCMs to concisely model SNP dependences, showing that the multiple layers of the model can take into account different LD degrees and haplotype diversity; (vi) finally, we present a thorough study focused on both scalability and impact of adjustment of the input parameters of CFHLC algorithm.

As a prerequisite to further understanding, Section Preliminaries provides an informal definition of Bayesian networks, focusing on latent class models and hierarchical latent class models. Then the Section dedicated to the state of the art first points out the few anterior works devoted to HLCM learning in general. This section ends with a short review of the few attempts to implement probabilistic graphical models for the specific purpose of LD modeling. Section Methods motivates the modeling of LD through FHLCMs and informally describes such models. Then, the focus is set on the general outline of the method proposed for FHLCM learning. The next Section depicts the sketch of algorithm CFHLC. The last Section is dedicated to experimental results and discussion. In this Section, we first test and discuss the ability of FHLCMs to accurately represent the haplotype cluster structure of genetic data. Then, we compare our algorithm to other methods with respect to faithfulness in LD modeling and data dimension reduction. We end the Section with a thorough study centered on scalability and influence of the input parameters of the CFHLC algorithm.

## Preliminaries

**Figure 1:**
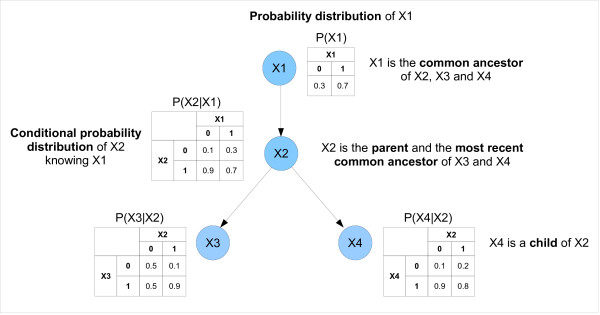
**Illustration of key terms specific to probabilistic graphical models**. The specific key terms illustrated below are the following: probability distribution, conditional probability distribution, common ancestor, most recent common ancestor, child and parent.

From now on, we will restrain the study to discrete and finite variables (either observed or latent). For readers that are not familiar with PGMs, Figure 1 clarifies the meaning of specific key terms used hereafter.

Bayesian networks are probabilistic graphical models. They are defined by a directed acyclic graph (DAG), *G*(*X*, *E*), and a set of parameters, *θ*. The set of nodes X = {*X*_1_, ..., *X_n_*} represents *n *random variables and the set of edges *E *captures the conditional dependences between these variables (*i.e*. the structure). The variables are either observed or latent. The set of parameters *θ *is a matrix of conditional probability distributions $\theta_{i}=\left[\mathbb{P}\left(X_{i}/Pa_{X_{i}}\right)\right]$ where $Pa_{X_{i}}$ denotes node *i*'s parents. If a node has no parent, then it is described by an *a priori *probability distribution. For further understanding, we now briefly introduce the concepts of marginal independence and conditional independence between two variables.

**Definition 1 ***The marginal independence between two variables X_i _and X_j _is defined referring to the joint distribution P*(*X_i _, X_j _*)*: P*(*X_i _, X_j _*) = *P*(*X_i _*) *P*(*X_j _*).

*A non-equality implies that X_i _and X_j _are marginally dependent*.

**Definition 2 ***More restrictive, the definition of conditional independence between two variables X_i _and X_j _given a subset of variables *S ⊆ ***X\***{*X_i _, X_j _*} *is the following: P*(*X_i _, X_j_*|S) *= P*(*X_i_*|S) *P*(*X_j_*|S).

*A non-equality implies that X_i _and X_j _are conditionally dependent given S*.

**Figure 2:**
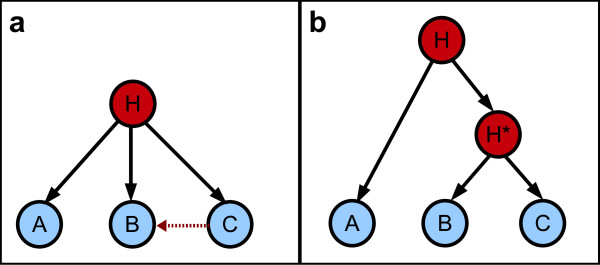
**Modeling of the local dependence between two nodes (a) Latent Bayesian network modeling the local dependence between B and C nodes. (b) Modeling of the local dependence between B and C nodes through a latent hierarchical model**. The light shade indicates the observed variables whereas the dark shade points out the latent variables.

A latent class model (LCM) is a particular type of Bayesian network. It is defined as containing a unique latent variable connected to each of the observed variables. The latent variable simultaneously influences all observed variables and hence renders them dependent. In the LCM framework, an underlying assumption, called local independence (LI), states that the observed variables are pairwise independent, conditional on the latent variable [23]. The intuition behind LI is that the latent variable is the only explanation for the dependences between observed variables. However, this assumption is often violated for observed data. To tackle this issue, HLCMs were proposed as a generalization of LCMs. HLCMs are tree-shaped BNs where leaf nodes are observed while internal nodes are not. In a Bayesian network, local dependence between variables may be modeled through the use of an additional latent variable (see Figure 2). On a larger scale, multiple latent variables organized in a hierarchical structure allow high modeling flexibility. Additional file 4 illustrates the ability of HLCMs to depict a large variety of relations encompassing local to higher-order dependences.

## State of the art

### HLC model learning

Various methods have been conceived to tackle HLCM learning. These approaches differ by the following points: (i) structure learning; (ii) determination of the latent variables' cardinalities; (iii) learning of parameters, *i.e. a priori *and conditional probabilities; (iv) scalability; (v) main usage.

As for general BNs, besides learning of parameters (*θ*), *i.e. a priori *and conditional probabilities, one of the tasks in HLCM learning is structure (S) inference. This task generally remains the most challenging due to the complexity of the search space. To address this issue, two main categories of HLCM learning methods have been developed. The first category, structural expectation maximization (SEM), successively optimizes *θ *conditional on S(θ|S) and S conditional on θ(S|θ). Amongst a few proposals, greedy search [24] and dynamic programming [25] were designed. They explore the space of possible graphs guided by a scoring function, such as the Bayesian information criterion (BIC) [26]. When using maximum likelihood estimation, the BIC score prevents model overfitting through a penalty term on the number of parameters in the model. As regards greedy search, the search space of HLCM structures can be visited through two operations: a structure in the neighborhood of the current structure may either result from the addition or the removal of latent nodes or from the addition or the dismissing of states, for existing nodes. In the other solution, implemented in Gerbil algorithm, dynamic programming discovers the best segmentation of a genomic region into blocks of contiguous SNPs. Then, for each previously learned block, an LCM is learned. Alternative approaches implement ascending hierarchical clustering (AHC), which provides clusters within which the SNPs are not necessarily contiguous. In the following, we will use the terms "blocks" and "clusters" to distinguish between these two possibilities. Relying on pairwise dependence strength, Wang and co-workers first build a binary tree; then they apply regularization and simplification transformations which may result in subsuming more than two nodes through a latent variable [27]. Hwang and collaborators' approach confines the HLCM search space to binary trees augmented with possible connections between siblings (nodes sharing the same parent into immediate upper layer) [28]. To construct the tree, they design an AHC strategy. First, a partition of the observed variables into clusters of size 2 is performed, based on a mutual information criterion. Any such cluster then defines a new LCM (thus a new LV) in the upper layer under construction. Second, the parameters of each LCM are learned. Thus missing values of LVs can be imputed. Therefore these LVs can be considered as observed variables for the next step. A tree is completed through the iteration of these two steps (partitioning, missing value imputation).

Parameter learning requires the determination of the LVs' cardinalities, *e.g*. the number of possible states (or classes) for each LV. A simple method is to arbitrarily set a small value for the cardinality. Following this idea, Hwang and collaborators constrain LVs to binary variables. This method is very fast but presents several drawbacks: on the one hand, a too small cardinality can lead to a loss of information in the process subsuming child variables into a unique LV; on the other hand, a too large cardinality can entail model overfitting and heavy computational burden. Wang and co-workers propose a regularization step to reduce the cardinality of an LV *Y*, knowing the cardinality of its neighbor variables $Z_{i}:|Y|=\frac{\Pi_{i=1}^{k}|Z_{i}|}{max_{i=1}^{k}|Z_{i}|}$. Other authors use a greedy search approach, starting with a preset value and incrementing or decrementing it to meet an optimal criterion. The latter method has the drawback of entailing computational overload because it runs several steps of the expectation maximization (EM) algorithm, implementing an iterative procedure.

Usually, the EM algorithm is used for parameter learning in the presence of LVs or missing data, but it is computationally expensive and does not guarantee that the global optimum will be reached. To speed up the EM process, Hwang and collaborators implemented a heuristic based on partial imputation of binary LVs' missing values. Thus, the EM algorithm is actually run on partially imputed data. For an LCM containing two child variables *Y_j _*and *Y_k_*, the heuristic is the following: all individuals showing the most probable configuration of {*Y_j_, Y_k_*} are assigned an LV value of 0. Similarly, the individuals characterized with the second most probable configuration are assigned an LV value of 1. To avoid getting trapped in local optima while running the EM algorithm to learn a set of latent models, other authors adapted a simulated annealing approach [16].

Hwang and co-workers' approach is the only one we are aware of that succeeds in processing high-dimensional data: in an application dealing with a microarray dataset, more than 6000 genes have been processed for around 60 samples. To the best of our knowledge, no running time was reported for this study. Nevertheless, the twofold binarity restriction (binary tree, binary LVs) and the lack of control for information decay as the level increases are severe drawbacks to reach our aims: *i.e*. to achieve realistic SNP dependence modeling and perform subsequent association study with sufficient power.

### Graphical models for LD modeling

To address LD modeling through a probabilistic graphical model framework, various models were proposed: hidden Markov models (HMM), Markov random fields and Bayesian networks with or without latent variables. HMMs represent simple but efficient models to partition a SNP sequence into blocks, because no structure learning step is required [29] and the latent states may represent common haplotypes. Verzilli and co-workers modeled SNP dependences using Markov random fields [12]. They designed an MCMC (Markov Chain Monte Carlo) method to sample over the space of possible graphs while exploiting prior biological knowledge. Their approach allows to discover cliques of dependent SNPs, to further allow the identification of causal relations between markers and the disease status indicator. To implement a tractable method for genome-wide data, Verzilli and co-workers reduce the space of possible graphs by specifying a maximal physical distance between SNPs belonging to the same clique, as well as a maximal size of 8 SNPs for any clique. In the family of Bayesian networks without LVs, HaploBlock implements a statistical model of haplotype block variation [13]. This model's advantage lies in integrating population genetics concepts such as recombination hotspots, bottleneck, genetic drift and mutations. Another method, BNTagger, was developed for SNP tag selection; it exploits conditional independence between variables [14]. To learn the structure, BNTagger implements a greedy search with random restarts; then it determines a subset of independent and highly predictive SNPs. The two latter methods were only tested on a small number of SNPs (less than 1000) and the authors reported running times of 40 *h *for 97 SNPs [13] and between 2 and 4 *h *for only 52 SNPs [14]. Thus, these methods do not seem fitted to GWAS data processing. Regarding the family of Bayesian networks with LVs, Nefian modeled SNP dependences through embedded Bayesian networks. Her model is indeed a set of LCMs augmented with SNP-SNP dependences and LV-LV dependences [15]. To learn the model, the SNP data sequence is split into contiguous windows of fixed common size. Then, for each window, an LCM is created. The lack of flexibility of the SNP partitioning method used remains a severe draw-back. Zhang and Ji also proposed to model LD through a set of LCMs, using an SEM strategy [16]. Their method does not require splitting the sequence into fixed-size windows. Nevertheless, the number of LCMs has to be specified. As far as we know, no execution times were reported for the two latter approaches when run on high-dimensional data.

Other methods are based on regularization, such as the graphical Lasso [30], and have been applied to learning sparse PGMs for proteomics or gene expression studies, whose data dimensionality is high (around 5000 variables) but lower than that of genome-wide data (above 100000 variables). The basic idea is to consider that the observations follow a multivariate Gaussian distribution with mean µ and covariance matrix Σ (graphical Gaussian model). If the *ij^th ^*partial correlation coefficient of the precision matrix Σ^-1 ^equals zero, variables *i *and *j *are conditionally independent, given the other variables. The use of Lasso aims at restraining the learning task to sparse PGMs through finding a least-square solution under the following constraint: ∑_ν_|*β_ν_*| ≤ *t*, meaning that the sum, over the whole variable set, of the absolute values of the regression coefficients *v *has to be inferior or equal to a constant *t*. This Lasso-based approach has been extended to the case where it is reasonable to assume that the variables can be clustered into groups sharing similar correlation patterns (corresponding to underlying biological modules in gene expression) and where sparse block-structured precision matrices are estimated [31].

To our knowledge, Verzilli *et al'*s method is the only one whose tractability regarding GWAS data is known. However, in practice, their MRF modeling reveals a drawback. The LD is modeled through cliques containing a maximum number of 8 SNPs, whereas, generally, several tens or hundreds of SNPs may be dependent. Furthermore, no dependences between cliques are taken into account. In contrast, BNs with LVs offer a crucial advantage over other models: they provide synthesizing variables useful to reduce data dimensionality. However, when the number of variables exceeds several hundreds, implementing the SEM approach for LD modeling leads to prohibitive computational burden. When dealing with genome-wide data, the imperious requirement for tractability leads us to choose a hierarchical clustering approach, in the line of Hwang and co-workers.

## Methods

### Motivation of the FHLC model for GWASs

The HLCMs offer several advantages for GWASs. First, beside data dimensionality reduction, they allow a simple test of direct dependence between an observed variable and a target variable such as the phenotype, conditional on the latent variable, parent of the observed variable. Note that the phenotype variable is not included in the HLCM. In the context of GWASs, this test helps find the markers which are directly associated with the phenotype, *i.e*. causal markers, should there be any. Second, HLCMs can deal with the fuzzy nature of LD blocks. Indeed, HLCMs can take into account various degrees of LD strength between any two SNPs, depending on the height of their lowest common LV node ancestor in the tree. Thirdly, the hierarchical structure allows zooming in through narrower and narrower regions in search for a stronger association with the disease, thus offering a data mining tool. This zooming process ends pointing out the potential markers of interest. Finally, the latent variables may be interpreted in terms of biological meaning. For instance, in the case of haplotypes, that is, phased genotypes, the latent variables are likely to represent the so-called haplotype block structure of LD. To a certain extent, an LV might be interpreted as the shared ancestry of the haplotypes defined by the observed variables, namely, the contemporary haplotypes of the tree rooted in the LV. Each state of an LV may represent a group of similar haplotypes. In the situation of limited ancestral recombination, similar haplotypes tend to share recent common ancestry. Although this situation is not guaranteed along the genome, it is very likely for low-level LVs, since they are expected to cover very small genomic regions showing strong LD. Thus the directed edges, *LV → SNP*, can represent causal effects and provide a biological sense. Besides, it has to be noted that when the latent variables capture dependences between distant SNPs (or distant groups of markers), they can be viewed as population structure.

However, SNP dependences would better be more wisely modeled through a forest of HLCMs. In the case of a forest, higher-order dependences are captured only when relevant, *i.e *when meeting a strength criterion. Therefore, FHLCMs allow to model a larger set of configurations than HLCMs do. Typically, an HLCM is limited to represent clusters of close dependent SNPs. Actually, in this model, variables are constrained to be dependent upon one another, either directly or indirectly. Consequently, HLCMs cannot account for potential independence between groups of distant SNPs or SNPs located on different chromosomes. But realistic modeling requires a more flexible framework. For instance, the LD plot of the 2 Mb sequence shown in Additional file 5 reveals that the greatest part of LD is observed between SNPs in vicinity. LD rarely exceeds 500 kb between SNPs.

**Figure 3:**
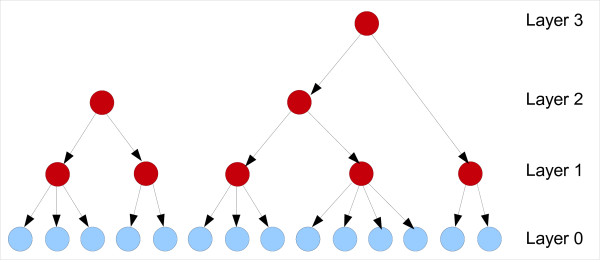
**A forest of hierarchical latent models**. This forest consists of two trees, of respective heights 2 and 3. The light shade indicates the observed variables whereas the dark shade points out the latent variables.

An FHLCM consists of a directed acyclic graph (DAG) also called the structure whose nonconnected components are trees, and of *θ*, the parameters (further defined). Figure 3 illustrates a possible structure for an FHLCM.

### Principle of FHLC model construction

Our method can process both genotypic (unphased) and haplotypic (phased) data. It takes as an input a matrix $D_{X}$ defined on a finite discrete domain, say {0, 1, 2} for unphased SNPs or {0, 1} for phased SNPs, describing *n *individuals through *p *variables X = {X_1_, ..., X*_p_*}. Algorithm CFHLC yields an FHLCM, that is a forest structure and *θ*, the parameters of a set of *a priori *distributions and local conditional distributions allowing the definition of the joint probability distribution. Two search spaces are explored: the space of directed forests and the probability space. In addition, the whole set of latent variables *H *of the FHLCM is output, together with the associated imputed data matrix.

**Figure 4:**
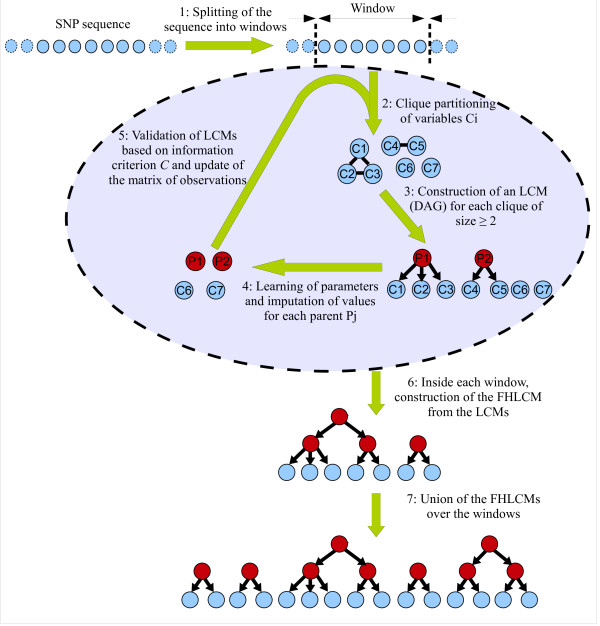
**Schema of the CFHLC algorithm**. The light shade indicates the observed variables whereas the dark shade points out the latent variables.

To handle high-dimensional data, our proposal combines two strategies. The first strategy splits up the genome-scaled data into contiguous regions. In our case, splitting into (large) windows is not a mere implementational trick; it satisfies biological grounds: the overwhelming majority of dependences between genetic markers (including higher-order dependences) is observed for close SNPs. The user interested in taking into account long-range LD due to the presence of population structure will be faced with the following choices: (i) adjusting the window size, relying on biological background defining the maximum physical distance between SNPs in long-range LD (*e.g*. 500 *kb *or 1 *Mb*); (ii) slightly diminishing the density of the studied SNP sequence. A combination of these two approaches may be more convenient. Then, an FHLCM is learnt for each window in turn. Within a window, subsumption is performed through an adapted AHC procedure: (i) at each agglomerative step, a partitioning method is used to identify clusters of variables; (ii) each such cluster is intended to be subsumed into an LV, through an LCM. For each LCM, parameter learning and missing data imputation (for the latent variable) are performed. A global schema of our method is presented in Figure 4.

Along with a hierarchy-based proposal of Hwang and collaborators [28] developed for gene expression studies, our method also implements data subsumption, meeting the two following additional requirements: (i) a more flexible thus more faithful modeling of underlying reality, (ii) a control of information decay due to subsumption.

### Node partitioning

**Figure 5:**
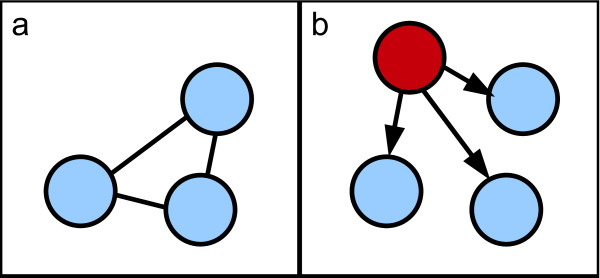
**Associating a latent variable to any clique of variables in the undirected graph of dependence relations (a) Three pairwise dependent variables (clique). (b) Latent model: the three variables depend on a common latent variable**. The dark shade indicates the latent variable designed to model the pairwise dependence between the three variables.

Following Martin and VanLehn [32], ideally, we would propose to associate a latent variable with any clique of variables in the undirected graph of dependence relations (see Figure 5). In the case when introducing an additional LV increases a scoring function such as the BIC score [26], the LCM is validated. However, searching for such cliques is an NP-hard task. Moreover, in contrast with these authors' objective, FHLCMs do not allow clusters to have more than one parent each: non-overlapping clusters are required for our purpose. Thus, an approximate method solving a *clique partitioning *problem when provided with pairwise dependence measures is relevant; the clique partitioning problem consists in finding the best partition of a graph into cliques.

An algorithm meeting this purpose has already been described in the literature: BenDor and co-authors designed CAST, a clique partitioning algorithm devoted to variable clustering [33]. They especially applied CAST for gene expression clustering. As an input, CAST requires a binary similarity matrix. The adaptation of CAST to our case is straightforward: the dependence measure between two SNPs, evaluated through mutual information, is used as a similarity measure. All mutual information values less than a threshold *t_MI _*are assigned a similarity value of 0, whereas the others are assigned a value of 1. As a threshold *t_MI _*, the median value (or another quantile value) of the mutual information matrix can be used. Then, the CAST algorithm constructs the clusters one at a time. The authors define the affinity *a*(*x*) of an element *x *to be the sum of similarity values between *x *and the elements present in the current cluster $C_{open}$. *x *is an element of high affinity if it verifies inequality *a*(*x*) ≥ *t_CAST_*|$C_{open}$|, where *t_CAST _*is a specified similarity threshold. Otherwise, *x *is considered an element of low affinity. To summarize, the algorithm alternates between adding high affinity elements to $C_{open}$ and removing low affinity elements from it. When the process stabilizes, $C_{open}$ is closed. A new cluster can be started.

### Determining cardinalities for latent variables

A steep task is choosing - ideally optimizing - the cardinality of each LCM's latent variable. This problem cannot be remedied using greedy search because of its intractability regarding high data dimensionality. Although the regularization method of Wang and collaborators has the advantage of being very quick (see Subsection HLC model learning), in our context, their method is impracticable. For instance, let us consider an HLCM learned from genome-wide data. The first layer of the HLCM contains the majority of the LVs in the model. In our case, an LV in the first layer can subsume more than 10 child OVs (*i.e*. SNPs). As the cardinality is the same for all OVs (3 possible genotypes: 0, 1, 2), the resulting cardinality of the LV after regularization remains generally very large. For example, for an LCM containing 10 OVs {*X*_1 _, ..., *X*_10 _} of cardinalities equal to 3, the cardinality of the LV *H *would be : |*H*| = 3^10^/3 = 3^9 ^= 19683. The simplest solution remains to arbitrarily set a small value for LV cardinalities, but it has several drawbacks (see Subsection HLC model learning). Instead of using an arbitrary constant value common to all latent variables, we propose that the cardinality be estimated for each latent variable through a function of the underlying cluster's size. The rationale for choosing this function is the following: the more child nodes a latent variable has, the larger the total number of possible combinations is for the values of the child variables and the larger also is the expected number of such combinations observed over all individuals (when the number of individuals is sufficiently high). Therefore, the cardinality of this latent variable should depend on the number of child nodes. Nonetheless, to keep the model complexity within reasonable limits, a maximum cardinality is fixed.

### Parameter learning and imputation

Parameter learning is carried out step by step, each time generating additional latent variables and imputing their values for each individual. At *i^th ^*step, this task simply amounts to performing parameter learning for as many LC models as there are clusters of variables identified. We recall that the nodes in the topology of an LCM are reduced to a unique root and leaves. Therefore, at *i^th ^*step, each LCM's structure is rooted in a newly created latent variable. When latent variables are the source nodes in a BN, parameter learning may be performed through a standard EM procedure. This procedure takes as an input the cardinalities of the latent variables and yields the probability distributions, that is, *prior *distributions for those nodes with no parents and distributions conditional to parents for the remaining nodes. After imputing the missing data corresponding to latent variables, new data are available to seed the next step of the FHLCM construction: latent variables identified through step *i *will be considered as observed variables during step *i *+ 1.

It has to be noted that designing an imputation method to infer the values of the latent variable for each individual is a matter for investigation. Once the *prior *and conditional distributions have been estimated for a given LCM, probabilistic inference in BNs may be performed. A straightforward way would consist in imputing the latent variable value for each individual as follows: $h*=argmax_{h}\left\{p\left(H=h/X_{j_{1}}=x_{j_{1}},X_{j_{2}}=x_{j_{2}},...,X_{j_{c}}=x_{j_{c}}\right)\right\}$. However, in the framework of probabilistic models, this deterministic approach is disputable. In contrast, a more convincing alternative will draw a value *h *for latent variable *H*, knowing the probabilities $p\left(H=h/X_{j_{1}}=x_{j_{l}},X_{j_{2}}=x_{j_{2}},...,X_{j_{c}}=x_{j_{c}}\right)$ for each individual.

### Controlling information decay

Conversely to Hwang and co-workers' approach, which mainly aims at data compression, information decay control is required: in step *i*, any candidate latent variable *H *which does not bear sufficient information about its child nodes must be invalidated. As a consequence, such child nodes will be seen as isolated nodes in step *i *+ 1.

Let us consider two variables *X *and *H*. Basically, the mutual information measures the difference of entropies between the independent model *P*(*X*) *P*(*H*) and the dependent model *P*(*X*|*H*) *P*(*H*): ℐ(X,H)=(ℋ(X)+ℋ(H))-(ℋ(X|H)+ℋ(H))=ℋ(X)-(ℋ(X|H). Therefore, the mutual information measures the dependence of the two variables. The larger the difference between entropies, the higher is the dependence. Now, let us consider a set of child variables X= {*X*_1_, *X*_2_, ..., *X_n_*} and the parent variable *H*. In our case, we want to compare the two models: *P(X*_1_) *P*(*X*_2_) ... *P*(*X_n_*) *P*(*H*) and *P*(*X*_1_|*H*) *P*(*X*_2_|*H*) ... *P*(*X_n_*|*H*) *P*(*H*). Thus, Δ, the difference of entropies between the two models is: $\left(\sum_{i=1}^{n}ℋ\text{(}X_{i}\text{)}+ℋ\text{(}H\text{)}\right)-\left(\sum_{i=1}^{n}ℋ\text{(}X_{i}|H\text{)}+ℋ\text{(}H\text{)}\right)=\sum_{i=1}^{n}\left(ℋ\text{(}X_{i}\text{)}-ℋ\text{(}X_{i}|H\text{)}\right)=\sum_{i=1}^{n}ℐ\text{(}X_{i}|H\text{)}$. Δ corresponds to the sum of mutual information values over all LCM's edges.

Normalization through entropy and averaging are performed to provide a more intuitive criterion: $C=\frac{1}{S_{H}}\sum_{i\;\in \;cluster\left(H\right)}\frac{ℐ\left(X_{i,}H\right)}{\min\left(ℋ\left(X_{i}\right),ℋ\left(H\right)\right)}$, with *S_H _*the size of *cluster *(*H*). C represents the average percentage of information captured by the LV with respect to its child variables.

## Algorithm

The sketch of CFHLC is presented in Algorithms 1 and 2. The user may tune seven parameters. Window size *s *specifies the number of contiguous SNPs - or variables - spanned per window. The aforementioned criterion *C *is meant to estimate information decay, thus allowing information dilution to be constrained to a minimal threshold *t*. Parameters *a*, *b *and *card_max _*participate in the calculus of the cardinality of each latent variable. Finally, parameter *PartitioningAlg *enables flexibility in the choice of the method dedicated to clustering highly-correlated variables into non-overlapping groups.

Within each window *i*, the AHC process is initiated from the first layer consisting of univariate models. Each such univariate model is built for any observed variable in the set $W_{i}$ (lines 6 to 8). The AHC process stops if each cluster identified is reduced to a singleton (line 13) or if no cluster of size strictly greater than 1 could be validated (line 31). Each cluster containing at least two nodes is subject to LCM learning (lines 19 and 20) followed by validation (line 23 to 28). In order to simplify the FHLCM learning, the cardinality of the latent variable is estimated as an affine function of the number of variables in the corresponding cluster (line 19). Algorithm *learn_latent_class_model *is plugged into this generic framework (line 20). After validation through threshold *t *(lines 23 and 24), the LCM is used to enrich the FHLCM associated with the current window (line 25): a specific merging process links the additional node corresponding to the latent variable to its child nodes, themselves already present in the FHLCM structure under construction; the *prior *distributions of the child nodes are replaced with distributions conditional on the latent variable. The newly created latent variable, $L_{j_{k}}$, is added to the set of latent variables, whereas its imputed values, $D\left[L_{j_{k}}\right]$, are stored (line 26). In $W_{i}$, the variables in $C\ell_{j_{k}}$ are now replaced as a whole with the corresponding latent variable; data matrix $D\left[W_{i}\right]$ is updated accordingly (line 27). In contrast, the nodes in unvalidated clusters are kept isolated for the next step. Finally, the collection of forests, DAG, is successively augmented with each forest built within a window (line 36). In parallel, due to assumed independence between windows, the joint distribution of the final FHLCM is merely computed as the product of the distributions associated with the windows (line 36).

INPUT:

X, a set of *p *variables (*X *= *X*_1_; ...; *X_p_*),

$D_{X}$, the corresponding observations for *n *individuals,

**s **, a window size,

C, a criterion designed to estimate information decay while building the FHLCM,

**t**, a threshold used to constrain information dilution, based on criterion C,

*PartitioningAlg*, an algorithm dedicated to partition a set of variables into non-overlapping clusters of variables,

**a**; **b **and **card_max_**, parameters used to estimate the cardinality of latent variables.

OUTPUT:

*DAG *and *θ*, respectively the DAG structure and the parameters of the FHLCM constructed,

*L*, the whole set of latent variables identified through the construction (*L *= {*L*_1,..., _*L_m_*}), $D_{L}$, the corresponding data imputed for *n *individuals.

1: *nbw← p/s */* computation of the number of contiguous windows */

2: *DAG ←*∅; *θ ← *∅; *L← *∅; *D_L_*← ∅

3:

4: **for ***i *= 1 **to ***nbw*

5:    /* processing of layer 0 */

6:    $W_{i}\leftarrow \left\{X_{\left(i-1\right)\times S+1},...,X_{i\times S}\right\};D\left[W_{i}\right]\leftarrow D\left[\left(i-1\right)\times S+1:i\times S)\right]$

7:    $\left\{\cup_{j\in W_{i}}DAG_{univ_{j}},\cup_{j\in W}{}_{{}_{i}}\theta_{univ_{j}}\}\leftarrow learn\_univariate\_models(W_{i}\right)$

8:    $DAG_{i}\leftarrow \cup_{j\in W_{i}}DAG_{univ}{}_{{}_{j}};\theta_{i}\leftarrow \cup_{j\in W_{i}}\theta_{univ}{}_{{}_{j}}$

9: 

10:    *step ← *1

11:    **while true**

12:    $\left\{C\ell_{1},\;...,\;C\ell_{\#C}\}\leftarrow partition(W_{i},D[W_{i}],PartitioningAlg\right)$

13:    **if **all clusters $C\ell_{q}$ are singletons **then break end if**

14: 

15:    $\left\{C\ell_{j_{1}},\;...,C\ell_{j_{\#C_{2}}}\right\}\leftarrow identify\_clusters\_of\_size\_strictly\_greater\_than\_one(C\ell_{1},\;...,C\ell_{\#C})$

16:    *nbValidClusters *← 0

17: 

18:    **for **k = 1 **to **$\#C_{2}$

19:    *card _LV _*← *min*(*a *× *number_of_variables *($C\ell_{j_{k}}$) + *b*; *card_max_*)

20:    {*DAG_jk_*, *θ_jk_*, *L_jk_*, $\left\{DAG_{j_{k}},\theta_{j_{k}},L_{j_{k}},D\left[L_{j_{k}}\right]\right\}\leftarrow learn\_latent\_class\_model\left(C\ell_{j_{k}},D\left[CL_{j_{k}}\right],card_{LV}\right)$

21: 

22:    /* validation of current cluster - see Subsection Controlling information decay */

23:    **if **$\left(C\left(DAG_{j_{k}},D\left[C\ell_{j_{k}}\right]\cup D\left[L_{j_{k}}\right]\right)\geq t\right)$

24:       *incr*(*nbValidClusters*)

25:       *DAG_i _*← *merge_structures*(*DAG_i_*, $DAG_{j_{k}}$); *θ_i _*← *merge_parameters*(*θ_i_*, $\theta_{j_{k}}$)

26:       $L\leftarrow L\cup L_{j_{k}};D_{L}\leftarrow D_{L}\cup D\left[L_{j_{k}}\right]$

27:       $D\left[W_{i}\right]\leftarrow \left(D\left[W_{i}\right]\backslash D\left[C\ell_{j_{k}}\right]\right)\cup D\left[L_{j_{k}}\right];W_{i}\leftarrow \left(W_{i}\backslash C\ell_{j_{k}}\right)\cup L_{j_{k}}$

28:    **end if**

29: **end for**

30:

31: **if **(*nbValidClusters *= 0) **then break end if**

32:

33: *incr*(*step*)

34: **end while**

35:

36: *DAG *← *DAG *∪ *DAG_i_*; *θ *← *θ *× *θ_i_*

37: **end for**

Algorithm 1: CFHLC

INPUT:

$C\ell_{u}$: a cluster containing at least two nodes,

$D\left[C\ell_{u}\right]$: the corresponding observations for *n *individuals,

*card_LV_*: the cardinality of the latent variable to be created.

OUTPUT:

a latent class model described by:

*DAG_u _*and *θ_u_*, respectively the structure and the parameters of the latent class model,

*L_u_*, a latent variable,

$D\left[L_{u}\right]$, the data imputed for the latent variable (for *n *individuals).

1: *Lu *← *create_latent_variable*()

2: *DAG_u _*← *build_structure_of_latent_class_model*(*L_u_*, $C\ell_{u}$)

3: *θ_u _*← *run_standard_EM*(*DAG_u_*, $D\left[CL_{u}\right]$, *card_LV_*)

4: $D\left[L_{u}\right]$ ← *impute_data*(*θ_u_*, $D\left[CL_{u}\right]$)

Algorithm 2: learn latent class model

## Experimental results and discussion

### Implementation

Algorithm CFHLC has been developed in C++, relying on the ProBT library dedicated to BNs http://bayesian-programming.org. We have plugged into CFHLC a C++ implementation of CAST based on the original implementation provided in JAVA by Ben Fry http://benfry.com/clustering/. Regarding the visualization of the DAGs, the software Tulip http://tulip.labri.fr/TulipDrupal/ was chosen, meeting both high representation quality and compactness requirements. CFHLC was run on a standard PC (3.8 GHz, 3.3 GB of RAM).

### Experimental protocol

The performance of the FHLCM-based method is evaluated using real phased and unphased genetic data, on the one hand, and simulated phased and unphased genetic data on the other hand.

For real data analysis, the well-known Daly *et al*. dataset [29], available at http://www-genome.wi.mit.edu/humgen/IBD5/index.html, was used. This dataset consists of 129 trios, each composed of two parents and one child. For each individual, 103 SNPs are genotyped in the 5*q*31 region and cover 617 *kb*. We only analyzed the child data.

Regarding simulated data, two well known programs were used: HAPGEN and HAP-SIMU. Using HAPGEN http://www.stats.ox.ac.uk/~marchini/software/gwas/hapgen.html, we generated 2000 unrelated individuals (*i.e*. 4000 haplotypes) for a several hundreds *kb *region containing around 20-30 SNPs. The haplotypes used as references come from the HapMap phase II and concern U.S. residents of northern and western European ancestry (CEU) http://hapmap.ncbi.nlm.nih.gov/. Five sequences showing variable LD degrees (*median*(*r*^2^)) ranging from 0.007 to 0.5 were generated.

HAPSIMU http://l.web.umkc.edu/liujian/ was used to simulate genotypes with simulation parameters described in Additional file 6. Three sample sizes were chosen with respect to the number of observed variables: 1 *k*, 10 *k *and 100 *k *SNPs (in all cases, the number of individuals was set to 2000). For each sample size, twenty benchmarks were generated. For these experimentations, imputation of LVs' values was achieved by assigning the most probable values given the observations. A drawback of this method is the loss of probabilistic relation between a variable and its parent variable. A definite advantage lies in its running in around half the time required by imputation through simulation (results not shown).

### LD modeling

#### Real data

Regarding the Daly benchmark, our aim was to evaluate how the forest obtained keeps up with the real structure of the biological data. Moreover, the CFHLC algorithm was compared to four other methods.

**Figure 6:**
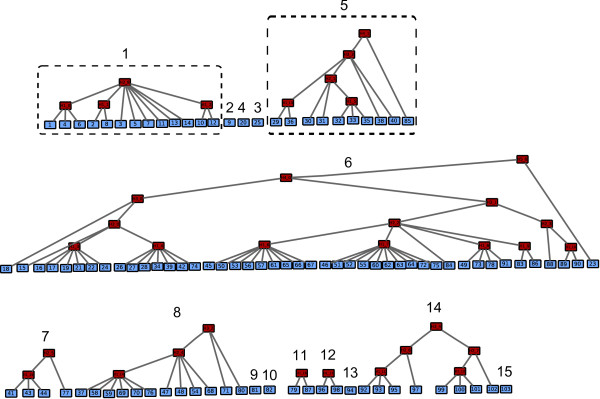
**Directed acyclic graph of the FHLC model learned for haplotypes (phased genotypes) of Daly *et al*.'s dataset**. The light shade indicates the observed variables whereas the dark shade points out the latent variables. Observed variables are numbered from 1 to 103 whereas latent variables are denoted "*Hℓ_i*" where *ℓ *specifies the layer number and *i *enumerates the different variables belonging to a same layer. We recall that in any FHLCM graph, edges are directed from top to bottom. *a *= 0.2, *b *= 2, *card_max _*= 20, *t_CAST _*= 0.95, *t_MI _*= *quantile_MI_*(0.95), *t *= 0.3 (for CFHLC parameter description, see Section Algorithm).

**Figure 7:**
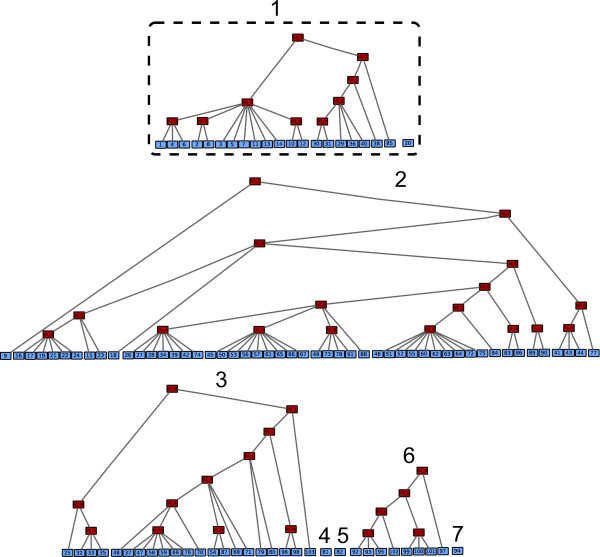
**Directed acyclic graph of the FHLC model learned for unphased genotypes of Daly *et al*.'s dataset**. For node nomenclature, see Figure 6. We recall that in any FHLCM graph, edges are directed from top to bottom. *a *= 0.2, *b *= 2, *card_max _*= 20, *t_CAST _*= 0.95, *t_MI _*= *quantile_MI _*(0.95), *t *= 0. 3 (for CFHLC parameter description, see Section Algorithm).

We learned FHLCMs on both haplotype (phased) and genotype (unphased) data. The corresponding graphs are displayed in Figures 6 and 7, respectively. Globally, the two graphs are similar: most of SNPs which are connected through an LV in the haplotype-data graph (HDG) are also connected through an LV in the genotype-data graph (GDG), *e.g*. SNP1, SNP4 and SNP6. Moreover, a substantial part of these SNPs share a common parent in both graphs: for instance, in both HDG and GDG, we observe that SNP61 and SNP65 are linked by an LV belonging to layer 1. Thus, learning FHLCM from genotype data instead of haplotype data leads to similar hierarchical structures. However, on average, we observe that the SNPs in the GDG are more connected: 8 and 15 connected components are identified in the HDG and the GDG, respectively. For example, the two framed trees 1 and 5 in the HDG of Figure 6 are linked by a high-level LV in the GDG of Figure 7 (see tree 1).

**Figure 8:**
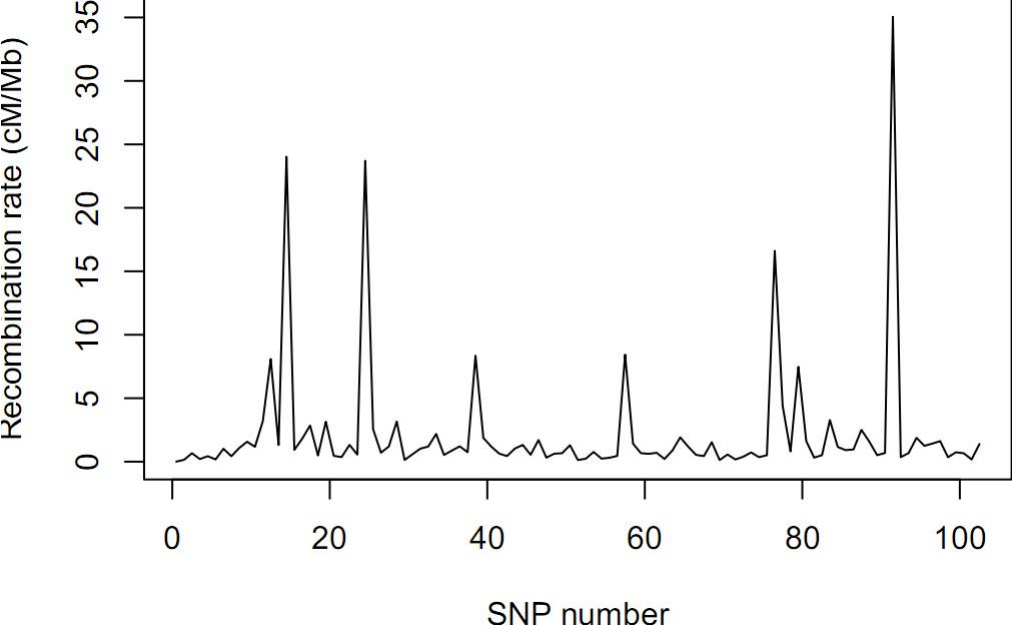
**Recombination rates (cM/Mb) inferred with software PHASE v2.1 for phased haplotypes of Daly *et al*.'s dataset**.

We expect that the FHLCMs' graphs will reflect the "haplotype block structure": large blocks of correlated contiguous SNPs separated by recombination hotspots. First, we observe that the physical position of SNPs influences their connection, since close SNPs tend to be linked by an LV belonging to a low layer, whereas distant SNPs are generally connected by a high-level LV. However, strong dependences between distant SNPs are also observed, *e.g*. between SNP26 and SNP74 or SNP49 and SNP91 (see Figure 6, tree 6 and Figure 7, tree 2). This characteristic reveals that the LD structure is not only dominated by spatial effects and justifies our haplotype cluster approach (instead of the standard haplotype block approach). In addition, the graphs interestingly show trends consistent with biological reality, that is the variation of the recombination rates inferred by software PHASE v2.1 [34] along the studied sequence (see Figure 8). Indeed, most of subtrees rooted in low-level LVs cover regions with low recombination rates (RR). More than 68% and 94% of LVs from layer 1 cover chromosomic segments showing RRs below 4 *cM*/*Mb *and 9 *cM*/*Mb*, respectively. The same tendency is observed for more than 44% and 66% of LVs from layer 2, respectively. These results show the relevance of (partly) interpreting low-level LVs as haplotype shared ancestry when CFHLC's input is haplotype data.

**Figure 9:**
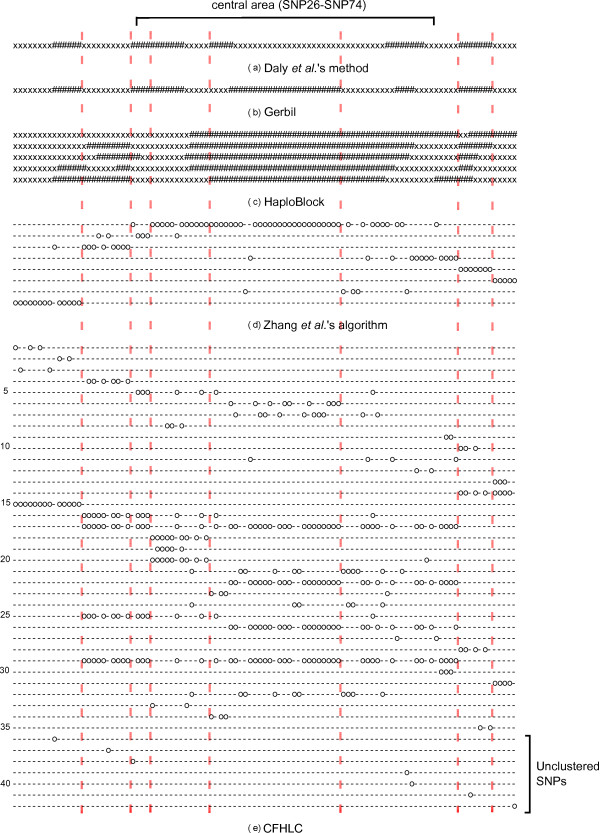
**Comparison of the outputs of five methods devoted to linkage disequilibrium modeling, for Daly *et al*.'s dataset**. Partitions of contiguous SNPs (blocks) inferred by (a) Daly *et al*.'s method, (b) Gerbil software and (c) HaploBlock. Subfigure (c) displays five different outputs produced by non-deterministic software HaploBlock. Blocks are represented by alternating sequences of x and #. Partitions of non contiguous SNPs (clusters) inferred by (d) Zhang *et al*.'s algorithm and (e) CFHLC algorithm. Subfigure (d) shows a partition of SNPs whereas Subfigure (e) displays a hierarchical clustering. Symbol o in *i^th ^*row and *j^th ^*column indicates that the *j^th ^*SNP belongs to the *i^th ^*cluster. Dotted lines highlight common trends between the five methods. Parameters for CFHLC algorithm: *a *= 0.2, *b *= 2, *card_max _*= 20, *t_CAST _*= 0.95, *t_MI _*= *quantile_MI _*(0.95), *t *= 0.3 (for CFHLC parameter description, see Section Algorithm).

We compared the structure obtained by CFHLC with those output by four other approaches: Daly *et al*.'s method [29], Gerbil [25], HaploBlock [13] and Zhang *et al*.'s algorithm [16]. All these methods were detailed in Section State of the art. The three former methods partition the sequence into blocks of contiguous SNPs. In contrast, the latter algorithm yields (non-overlapping) clusters of non-contiguous SNPs. We recall that CFHLC algorithm generates a hierarchical clustering of non-contiguous SNPs. In Figure 9, we compare the haplotype block- or cluster-structures obtained through all five methods aforecited. In spite of the fact that these methods differently tackle LD modeling, common trends emerge (see dotted lines in Figure 9). For instance, the last block identified by Daly *et al*.'s method, Gerbil and Zhang *et al*.'s algorithm (line 6) is also inferred by our algorithm in line 31. Slight differences are observed with the two first blocks resulting from Daly *et al*.'s method and Gerbil which only form one block for Zhang *et al*.'s algorithm (line 8) and CFHLC (line 15). Compared to other methods, most divergences with our algorithm remain in its unique ability to take into account the fuzzy delimitations of clusters. This is illustrated with the central area of the sequence (SNP26-SNP74), which actually presents two weak recombination hotspots (between SNP39 and SNP40, and between SNP58 and SNP59). Another difference with the other methods is the presence of "unclustered" SNPs, like SNP9, SNP20 and SNP25 in our model.

The running times, dimension reduction rates and entropy compression rates of all methods are reported in Table 1. Results show that Gerbil is the fastest algorithm tested, with a running time of 40 *s*. However, CFHLC and Zhang *et al*.'s algorithm, which learn more complex models (*i.e*. SNP clusters instead of SNP blocks), achieve their tasks in quite a reasonable time, 84 *s *and 168 *s*, respectively. Compared to others, HaploBlock is the slowest method, with a running time of 155 *mn*, due to the high complexity of learning models based on population genetics. For the three methods exhibiting a partition of contiguous SNPs, we defined the dimension reduction rate (DRR) as the ratio of the number of blocks to the number of SNPs. As regards Zhang *et al*.'s algorithm, the DRR was defined as the ratio of the number of clusters to the number of SNPs. In the case of CFHLC, we consider that the information of each FHLCM's tree can be synthesized by its root, providing the best dimension reduction. Therefore, in this case, the DRR is defined as the number of roots in the whole forest divided by the number of SNPs. HaploBlock generates the lowest number of blocks with an average of 6.8 (DRR value of 0.066), whereas Zhang *et al*.'s algorithm partitions the sequence in 8 clusters (DRR value of 0.078), and Daly *et al*.'s method and Gerbil both identify 11 blocks (DRR value of 0.107). CFHLC presents the lowest dimension reduction with 15 trees (DRR value of 0.146), due to the presence of 7 "unclustered" SNPs: SNP9, SNP20, SNP25, SNP81, SNP82, SNP94 and SNP103. As an alternative measure of compression, we defined the entropy compression rate (ECR) as the ratio of the sum of block (or cluster) entropies in a partition to the entropy, assuming no structure (*i.e*. the sum of individual SNP entropies). We observe a different ranking of the methods. We notice that CFHLC and Zhang *et al*.'s algorithm, which both learn cluster models, provide the best (*i.e*. lowest) ECR values (each around 0.23), whereas HaploBlock, Gerbil and Daly *et al*.'s method show ECR values of 0.241, 0.3 and 0.313, respectively. Regarding the ECR criterion, the comparatively better results obtained for the two cluster models are explained by the absence of the constraint for compulsory physical proximity between SNPs (as in blocks). Moreover, the ECR criterion does not penalize anymore the CFHLC algorithm, since the unclustered SNPs contribute relatively little to the overall information content.

**Table 1:** Comparison of running times, dimension reduction rates and entropy compression rates between CFHLC and other algorithms, for Daly *et al*.'s dataset: Daly *et al*.'s method [29], Gerbil [25], HaploBlock [13] and Zhang *et al*.'s algorithm [16].

Algorithm	Running time	Dimension reduction rates	Entropy compression rates
Daly *et al*.'s method	-	0.107	0.313
Gerbil	40 *s*	0.107	0.300
HaploBlock	158 *mn*	0.066	0.241
Zhang *et al*.'s algorithm	168 *s*	0.078	0.229
CFHLC	84 *s*	0.146	0.231

We ran the last three programs on a standard computer. As we had no access to Daly *et al*.'s software, we could only compare the dimension reduction rates and entropy compression rates calculated from their results with the dimension reduction rates and entropy compression rates obtained with the other methods.

**Figure 10:**
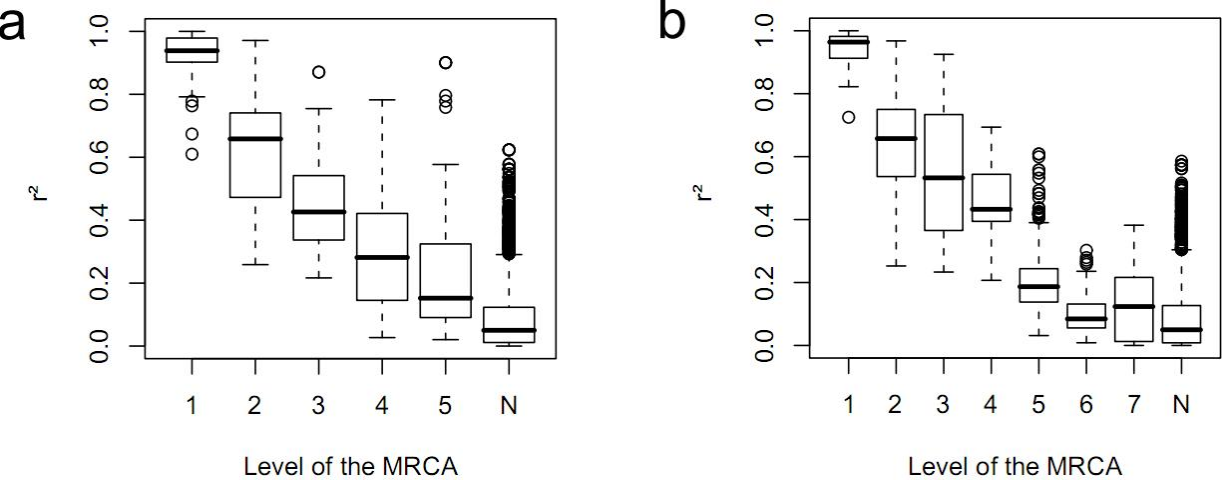
**Squared correlation coefficient of any pair of SNPs against the level of the most recent common ancestor (MRCA), for Daly *et al*.'s dataset**. (a) Phased data (b) Unphased data. N denotes the situation where the two SNPs considered do not belong to the same tree. *a *= 0.2, *b *= 2, *card_max _*= 20, *t_CAST _*= 0.95, *t_MI _*= *quantile_MI_*(0.95), *t *= 0.3 (for CFHLC parameter description, see Section Algorithm).

**Figure 11:**
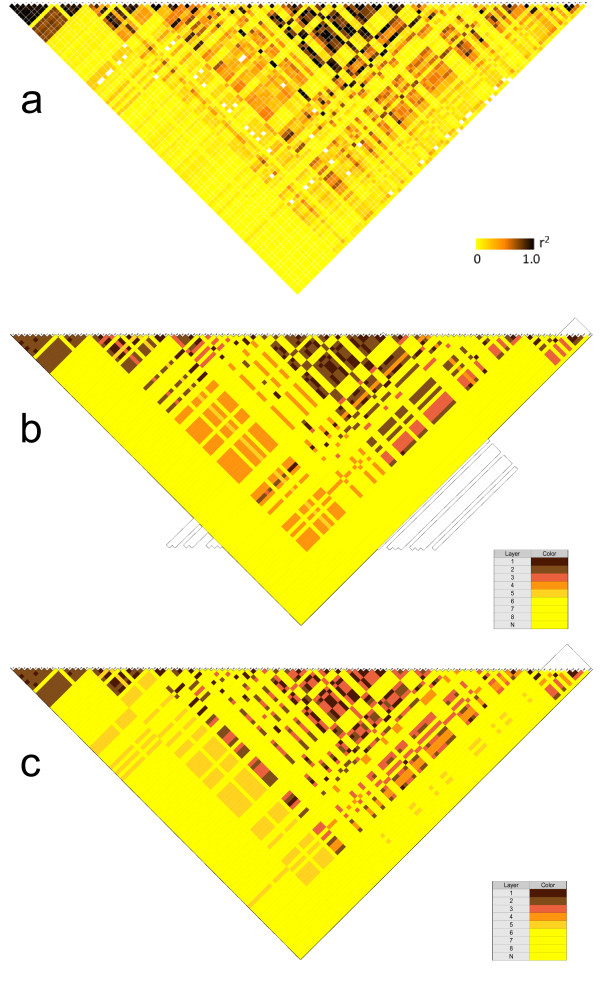
**LD plot *versus *matrix of MRCA levels. (a) LD plot (matrix of pairwise dependences between genetic markers - or linkage disequilibrium -) for the real data benchmark of Daly *et al*. (b) Triangular matrix of the MRCA levels learned from haplotype data. (c) Triangular matrix of the MRCA levels learned from genotype data. For any pair of SNPs, the MRCA is the most recent common ancestor**. The dataset consists of 103 SNPs in the 5*q*31 region; 129 individuals are described. This LD plot comes from [16]. As regards the two MRCA matrices, the color shade is all the darker as the MRCA level is high. N denotes the situation where the two SNPs considered do not belong to the same tree. *a *= 0.2, *b *= 2, *card_max _*= 20, *t_CAST _*= 0.95, *t_MI _*= *quantile_MI_*(0.95), *t *= 0.3 (for CFHLC parameter description, see Section Algorithm).

In Subsection Motivation of the FHLC model for GWASs, we argued that the multiple layers of an FHLCM can describe various degrees of LD strength. To analyze this property, we plotted the *r*^2 ^squared correlation coefficient of any pair of SNPs against the level of their most recent common ancestor (MRCA). Figure 10(a) and 10(b) show such plots drawn for haplotype and genotype data, respectively. Starting from values in the range [0.9-1.0], the *r*^2 ^correlation coefficient quasi-linearly decreases when the MRCA level increases. We conclude that the layered structure of the FHLCM faithfully reflects LD strength variety. These encouraging results lead us to visually compare the LD plot and the triangular matrices of the MRCA levels for haplotype (phased) and genotype (unphased) data, as presented in Figure 11. For this purpose, the same color code was used in the LD plot and the triangular matrix of MRCA levels. In the LD plot, the color (intensity) of each cell varies with the *r*^2 ^value. Since the median value of *r*^2 ^can be computed for each MRCA level, the color of each cell in the triangular matrix of MRCA levels is set, relying on the color scale used for the LD plot.

We expected to observe a correspondence between the three plots. The outstandingly clear correspondence demonstrates the ability of FHLCMs to accurately model multiple levels of LD strength: the overall majority of the LD plot dependences are also present in the MRCA level matrix. Interestingly, modeling from genotype data leads to quite good results compared to haplotype data.

**Figure 12:**
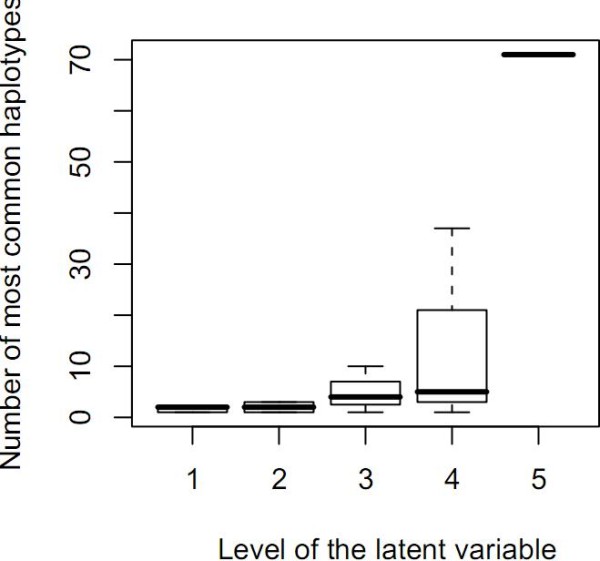
**Number of most common haplotypes against the latent variable's level, for Daly *et al*.'s dataset**. For any latent variable, observed haplotypes are defined by the observed variables, namely, the values for the leaves of the tree rooted in the latent variable. The set of the most common haplotypes is the smallest subset of observed haplotypes which covers at least 75% of the sample. Haplotype diversity is evaluated as the number of most common haplotypes observed at level l. *a *= 0.2, *b *= 2, *card_max _*= 20, *t_CAST _*= 0.95, *t_MI _*= *quantile_MI_*(0.95), *t *= 0.3 (for CFHLC parameter description, see Section Algorithm).

Haplotype diversity is generally very low within haplotype blocks or clusters. In our hierarchical model, haplotype diversity is expected to be all the larger within a cluster as the level of the LV subsuming this cluster is high. To check this point, we have relied on the clusters of Figure 9(e). For each LV, haplotype diversity has been calculated as the number of the most common haplotypes observed at level l of the tree rooted in this LV. Figure 12 plots the number of the most common haplotypes against the LV level. The plot shows that the haplotype diversity median remains very low (below 6) for the first four layers and dramatically increases to around 70 in the fifth layer. These results confirm our expectation relative to haplotype diversity in FHLCMs.

#### Simulated data

**Figure 13:**
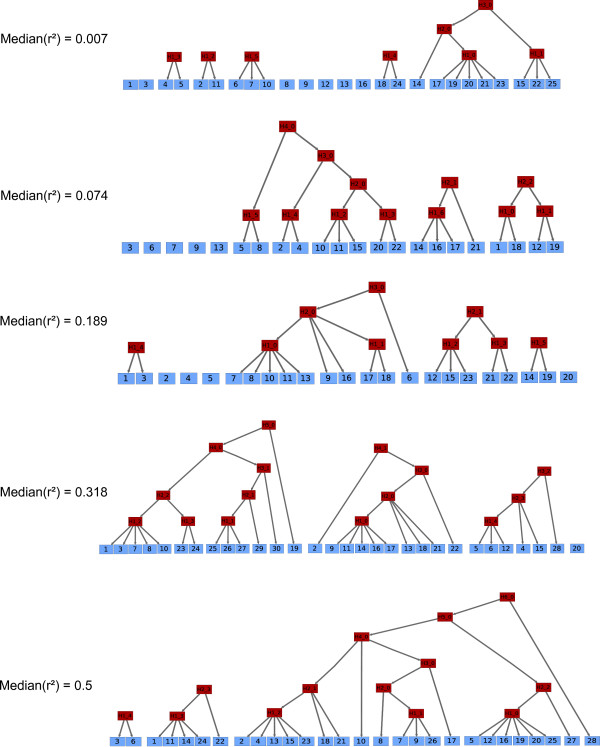
**Impact of LD degree on the construction of Forests of Hierarchical Latent Class models**. Five sequences showing variable LD degrees have been used to learn Forests of Hierarchical Latent Class Models. For display convention and node nomenclature, see Figure 6. We recall that in any FHLCM graph, edges are directed from top to bottom. *a *= 0.2, *b *= 2, *card_max _*= 20, *t_CAST _*= 0.95, *t_MI _*= *quantile_MI_*(0.95), *t *= 0.6 (for CFHLC parameter description, see Section Algorithm).

Finally, the impact of varying LD degrees was studied. For this purpose, we generated haplotype data with the HAPGEN software. Five sequences, showing variable LD degrees (*median*(*r*^2^)) ranging from 0.007 to 0.5, were used to learn FHLCMs. Figure 13 shows the forests obtained. The forests reveal that increasing LD degrees entails higher graph connectivity as well as a larger number of layers. Indeed, when *median*(*r*^2^) equals 0.007, 11 connected components are identified and the highest LV belongs to the third layer. Conversely, in the case when *median*(*r*^2^) is equal to 0.5, the forest is only composed of 3 connected components and the highest LV belongs to layer 6. Thus, we conclude that CFHLC can process sequences with various LD degrees and generate FHLCMs whose structures reliably reflect linkage disequilibrium.

### Scalability for GWASs

Scalability has been studied through the data simulated with HAPSIMU. In the hardest case (100 *k *SNPs), Additional file 7 shows that only 15 hours are required with a window size *s *set to 100. For the same dataset processed in the cases "*s *= 200" and "*s *= 600", running times are 20.5 *h *and 62.5 *h*, respectively. For the same number of OVs (100 *k*), Wang *et al*. report running times of about two months. Regarding the 10 *k *case, running times are 1.3 *h*, 2 *h *and 5.8 *h *for "*s *= 100", "*s *= 200" and "*s *= 600", respectively.

### Further analysis of CFHLC algorithm

Finally, many other experimentations are reported with their commentaries in additional files 8, 9, 10, 11, 12, 13, 14 and 15. Additional file 8 focuses on the impact of window size on running time. In additional file 9 data dimension reduction is evaluated from the distribution of LVs over the forest's layers. Additional files 10, 11, 12 and 13 study the impact of window size on the number of roots in the forest, the number of LVs, the number of layers and the distribution of LVs over the forest's layers, respectively. Additional files 14 and 15 analyze how information fades while the layer number increases.

An important result is that CFHLC can achieve a data dimensionality reduction of more than 80% of the number of observed variables (see Additional file 10). Regarding spatial complexity of CFHLC algorithm, the entire FHLCM does not require to be stored in RAM because each window can be saved on the hard disk.

## Conclusions

Our contribution in this paper is twofold: (i) a new framework has been described, which was tested on both real and simulated data and was proven able to concisely model LD and to reduce SNP data dimensionality; (ii) CFHLC, an algorithm dedicated to learn FHLCMs, has been shown to be efficient when run on genome-scaled benchmarks.

Compared to Verzilli and co-workers' works, our algorithm provides a more accurate modeling of LD and synthesizes genetic marker information through LVs. In addition, unlike Nefian or Zhang and Ji, our method does not require to specify the number of LCMs and can capture multiple levels of dependences, thus taking into account the fuzzy nature of LD. To our knowledge, our hierarchical method is the first one shown to achieve fast model learning for genome-scaled data sets, while maintaining satisfying information scores and relaxing the twofold binarity restriction of Hwang and collaborators' model (binary trees, binary latent variables). Hwang and collaborators' purpose is only data compression. We are faced with a more demanding challenge: to make a sufficiently powerful down-stream association analysis possible.

In discussing the biological interpretation of latent variables, we mentioned the potentiality of FHLCMs for population substructure description. In essence, using hierarchical models is highly appealing to take into account the long-range LD expected in substructured populations. However, this interesting use of such hierarchical models as FHLCMs is somewhat precluded by the technical necessity to partition the genome into small regions. As a first palliative, we indicated two strategies (adjusting the window size, diminishing the density) to cope with this current technical limitation. However, the strong expectation for faithful substructure modeling through FHLCMs advocates further efforts to clear the hurdle on path to realistic long-range LD modeling.

A bottleneck currently lies in the clique partitioning method chosen, which forbids window sizes encompassing more than 600 observed variables. In addition to investigating alternative partitioning methods, a lead to cope with this bottleneck may be to adapt the specific processings at the limits of contiguous windows or use overlapping windows.

In short, FHLCMs can be used to resolve several major problems in the GWASs' context. Beside flexible data dimension reduction through FHLCMs' LVs, fine mapping of causal SNPs is expected thanks to conditional independence properties encoded in such models. For instance, FHLCMs' LVs can be used to condition tests for independence between a SNP and the phenotype. Moreover, due to their hierarchical structure, FHLCMs represent an original and appropriate solution to study long-range LD in substructured populations, a recurring problematic in GWASs. Finally, genome-wide visualization of LD can be easily achieved with these models using a graph visualization tool and will provide an intuitive representation of *SNP *- *SNP *dependences as well as information synthesis through latent variables.

In this current version of CFHLC, when processing haplotype data, we were not interested in knowing the sequence of each ancestral haplotype. We just wanted to know from which ancestral haplotype (*i.e*. from which haplotype cluster) a contemporary haplotype comes. Nevertheless, it is feasible to infer the sequence of each ancestral haplotype using probabilistic inference.

Finally, although our modeling is designed for GWAS data, we emphasize that it could be applied to other data presenting spatial dependences between variables, in particular sequential data. Beyond this specific case, it would be interesting to assess the model's generality, in order to determine if it can be applied to generic graphical model learning.

## Authors' contributions

RM and CS both wrote the manuscript. RM proposed the modeling of the genetic data, the conception of a tractable algorithm and carried out the implementation and the experiments. CS designed the study, participated in its coordination and partook in the conception of the algorithm. PL participated in the design of the study, the conception of the algorithm, helped to improve the manuscript and provided indispensable feedback. All authors read and approved the final version of the manuscript.

## Supplementary Material

Additional file 1 **Direct and indirect associations between a genetic marker and the phenotype**. The figure included into this additional file illustrates the cases of direct and indirect associations between a genetic marker and the phenotype. Click here for file

Additional file 2 **Linkage disequilibrium plot for a simplified haplotype block structure**. The figure included into this additional file describes a standard representation of pairwise dependences between genetic markers. Click here for file

Additional file 3 **Linkage disequilibrium plot of a real 500 kb SNP sequence**. The figure presented in this additional file shows the linkage disequilibrium plot of a real 500 kb SNP sequence. Click here for file

Additional file 4 **Hierarchical latent class model**. The figure presented in this additional file depicts a hierarchical latent class model. Click here for file

Additional file 5 **Linkage disequilibrium plot of a 2 Mb SNP sequence**. The figure included in this additional file describes the linkage disequilibrium plot of a 2 Mb SNP sequence. Click here for file

Additional file 6 **Parameter value adjustment for the generation of simulated genotypic data through software HAPSIMU**. The table included in this additional file enumerates the values chosen for the parameters of software HAPSIMU. Click here for file

Additional file 7 **Average running time versus number of variables**. The figure presented in this additional file plots the running time of the CFHLC algorithm *versus *the number of SNPs in the dataset. Click here for file

Additional file 8 **Impact of window size on running time**. The figure presented in this additional file plots the running time of the CFHLC algorithm *versus *the window size. Click here for file

Additional file 9 **Number of variables per layer over the whole FHLC model**. The figure included in this additional file describes the average distribution of the variables over the layers (over 20 benchmarks). Click here for file

Additional file 10 **Impact of window size on the number of roots**. The figure included in this additional file depicts the impact of window size on the number of roots. Click here for file

Additional file 11 **Impact of window size on the number of latent variables**. The figure presented in this additional file shows the impact of window size on the number of latent variables. Click here for file

Additional file 12 **Impact of window size on the number of layers**. The figure presented in this additional file describes the impact of window size on the number of layers. Click here for file

Additional file 13 **Impact of window size of the number of latent variables per layer and on the ratio of the number of latent variables per layer to the total number of variables**. The two subfigures included in this additional file depict the impact of window size on the number of latent variables per layer on the one hand and the impact of window size on the number of latent variables per layer to the total number of variables, on the other hand. Click here for file

Additional file 14 **Impact of window size on scaled mutual information, per layer**. The figure presented in this additional file describes the impact of window size on scaled mutual information, per layer, over the whole FHLC model. Click here for file

Additional file 15 **Average scaled mutual information per layer over the whole FHLC model; impact of parameters *a *and *b***. The figure presented in this additional file shows the impact of parameters *a *and *b *on scaled mutual information, per layer, over the whole FHLC model. Click here for file

## References

[B1] Morris AP Cardon LR Handbook of statistical genetics, Whole genome association 2007 2 3 Wiley Interscience 1238 1263

[B2] Balding DJ A tutorial on statistical methods for population association studies Nature Genetics 2006 7 781 790 10.1038/nrg1916 16983374

[B3] International HapMap Consortium A second generation human haplotype map of over 3.1 million SNPs Nature 2007 449 7164 851 861 http://dx.doi.org/10.1038/nature06258 10.1038/nature06258 17943122 PMC2689609

[B4] DeWan A Klein RJ Hoh J Linkage disequilibrium and association mapping: analysis and applications, Linkage disequilibrium mapping for complex disease genes 2007 376 Humana Press 85 107 10.1007/978-1-59745-389-9_7 17984540

[B5] Patil N Berno AJ Hinds DA Barrett WA Doshi JM Hacker CR Kautzer CR Lee DH Marjoribanks C McDonough DP Nguyen BT Norris MC Sheehan JB Shen N Stern D Stokowski RP Thomas DJ Trulson MO Vyas KR Frazer KA Fodor SP Cox DR Blocks of limited haplotype diversity revealed by high-resolution scanning of human chromosome 21 Science (New York, N.Y.) 2001 294 5547 1719 1723 http://dx.doi.org/10.1126/science.1065573 10.1126/science.1065573 11721056

[B6] International HapMap Consortium The international HapMap project Nature 2003 426 6968 789 796 http://www.nature.com/nature/journal/v426/n6968/full/nature02168.html 10.1038/nature02168 14685227

[B7] Schaid DJ Evaluating association of haplotypes with traits Genetic Epidemiology 2004 27 348 364 10.1002/gepi.20037 15543638

[B8] Pattaro C Ruczinski I Fallin DM Parmigiani G Haplotype block partitioning as a tool for dimensionality reduction in SNP association studies BMC Genomics 2008 9 405 http://dx.doi.org/10.1186/1471-2164-9-405 10.1186/1471-2164-9-405 18759977 PMC2547855

[B9] Han B Kang H Seo M Zaitlen N Eskin E Efficient association study design via power-optimized tag SNP selection Annals of Human Genetics 2008 72 6 834 847 http://dx.doi.org/10.1111/j.1469-1809.2008.00469.x 10.1111/j.1469-1809.2008.00469.x 18702637 PMC2574965

[B10] Liang Y Kelemen A Statistical advances and challenges for analyzing correlated high dimensional SNP data in genomic study for complex diseases Statistics Surveys 2008 2 43 60 10.1214/07-SS026

[B11] Laramie JM Wilk JB DeStefano AL Myers RH HaploBuild: an algorithm to construct non-contiguous associated haplotypes in family based genetic studies Bioinformatics 2007 23 2190 2192 10.1093/bioinformatics/btm316 17586827 PMC2665175

[B12] Verzilli CJ Stallard N Whittaker JC Bayesian graphical models for genome-wide association studies The American Journal of Human Genetics 2006 79 100 112 10.1086/505313 PMC1474122 16773569

[B13] Greenspan G Geiger D High density linkage disequilibrium mapping using models of haplotype block variation Bioinformatics 2004 20 137 144 10.1093/bioinformatics/bth907 15262792

[B14] Lee PH Shatkay H BNTagger: improved tagging SNP selection using Bayesian networks Bioinformatics 2006 22 14 211 219 10.1093/bioinformatics/btl233 16873474

[B15] Nefian AV Learning SNP dependencies using embedded Bayesian networks IEEE Computational Systems, Bioinformatics Conference 2006

[B16] Zhang Y Ji L Clustering of SNPs by a structural EM algorithm International Joint Conference on Bioinformatics, Systems Biology and Intelligent Computing 2009 147 150

[B17] Freidlin B Zheng G Li Z Gastwirth JL Trend tests for case control studies of genetic markers: power, sample size and robustness Human Heredity 2002 53 146 152 10.1159/000064976 12145550

[B18] Hoggart CJ Whittaker JC De Iorio M Balding DJ Simultaneous analysis of all SNPs in genome-wide and resequencing association studies PLoS Genetics 2008 4 1 8 10.1371/journal.pgen.1000130 PMC2464715 18654633

[B19] Hahn LW Ritchie MD Moore JH Multifactor dimensionality reduction software for detecting gene-gene and gene-environment interactions Bioinformatics 2003 19 376 382 10.1093/bioinformatics/btf869 12584123

[B20] Schadt EE Lamb J Yang X Zhu J Edwards S Guhathakurta D Sieberts SK Monks S Reitman M Zhang C Lum PY Leonardson A Thieringer R Metzger JM Yang L Castle J Zhu H Kash SF Drake TA Sachs A Lusis AJ An integrative approach to infer causal associations between gene expression and disease Nature Genetics 2005 37 710 717 10.1038/ng1589 15965475 PMC2841396

[B21] Dixon AL Liang L Moffatt MF Chen W Heath S Wong KCC Taylor J Burnett E Gut I Farrall M Lathrop MG Abecasis GR Cookson WOC A genome-wide association study of global gene expression Nature Genetics 2007 39 1202 1207 10.1038/ng2109 17873877

[B22] Mourad R Sinoquet C Leray P Learning hierarchical Bayesian networks for genome-wide association studies19th International Conference on Computational Statistics (COMPSTAT) 2010 549 556

[B23] Zhang NL Hierarchical Latent Class models for cluster analysis The Journal of Machine Learning Research 2004 5 697 723

[B24] Zhang NL Kocka T Efficient learning of Hierarchical Latent Class models Proceedings of the 16th IEEE International Conference on Tools with Artificial Intelligence (ICTAI) 2004 585 593 full_text

[B25] Kimmel G Shamir R Gerbil: genotype resolution and block identification using likelihood Proceedings of the National Academy of Sciences of the United States of America 2004 102 158 162 10.1073/pnas.0404730102 15615859 PMC544046

[B26] Schwartz G Estimating the dimension of a model The Annals of Statistics 1978 6 2 461 464 10.1214/aos/1176344136

[B27] Wang Y Zhang NL Chen T Latent tree models and approximate inference in Bayesian networks Machine Learning 2006 32 879 900

[B28] Hwang KB Kim BH Zhang BT Learning hierarchical Bayesian networks for large-scale data analysis ICONIP 2006 670 679

[B29] Daly MJ Rioux JD Schaffner SF Hudson TJ Lander ES High-resolution haplotype structure in the human genome Nat Genet 2001 29 2 229 232 http://dx.doi.org/10.1038/ng1001-229 10.1038/ng1001-229 11586305

[B30] Friedman J Hastie T Tibshirani R Sparse inverse covariance estimation with the graphical Lasso Biostat 2008 9 3 432 441 http://dx.doi.org/10.1093/biostatistics/kxm045 10.1093/biostatistics/kxm045 PMC3019769 18079126

[B31] Marlin BM Murphy KP Sparse Gaussian graphical models with unknown block structure ICML '09: Proceedings of the 26th Annual International Conference on Machine Learning 2009 New York, NY, USA: ACM 705 712 http://dx.doi.org/10.1145/1553374.1553465

[B32] Martin J Vanlehn K Discrete factor analysis: learning hidden variables in Bayesian networks Tech. rep 1995 Department of Computer Science, University of Pittsburgh http://www.public.asu.edu/~kvanlehn/Not%20Stringent/PDF/94TR_JDM_KVL.pdf

[B33] Ben-Dor A Shamir R Yakhini Z Clustering gene expression patterns Proceedings of the third annual international conference on Computational molecular biology 1999 33 42 10.1089/106652799318274 10582567

[B34] Stephens M Scheet P Accounting for decay of linkage disequilibrium in haplotype inference and missing-data imputation American journal of human genetics 2005 76 3 449 462 http://dx.doi.org/10.1086/428594 10.1086/428594 15700229 PMC1196397

