# Supplementary material for: A hierarchical Bayesian network approach for linkage disequilibrium modeling and data-dimensionality reduction prior to genome-wide association studies
Source: BMC Bioinformatics. 2011 Jan 12;12:16. doi: 10.1186/1471-2105-12-16 (PMC3033325; doi:10.1186/1471-2105-12-16)
Supplement: Additional file 1 — Direct and indirect associations between a genetic marker and the phenotype. The figure included into this additional file illustrates the cases of direct and indirect associations between a genetic marker and the phenotype. [file 1471-2105-12-16-S1.PDF]

Direct and indirect associations between a genetic marker and the phenotype.

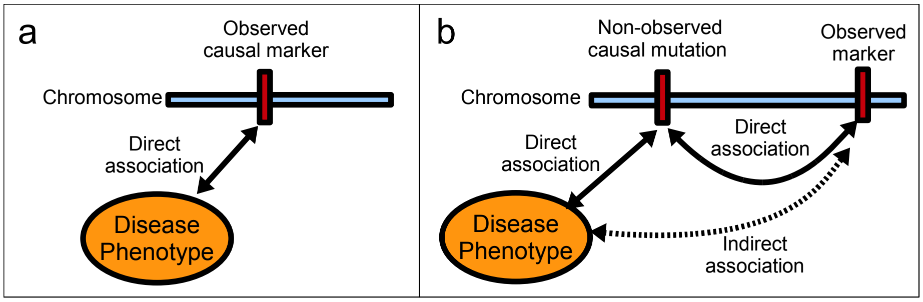

(a) Direct association between a genetic marker and the phenotype. (b) Indirect association between a genetic marker and the phenotype.
