# Supplementary material for: A hierarchical Bayesian network approach for linkage disequilibrium modeling and data-dimensionality reduction prior to genome-wide association studies
Source: BMC Bioinformatics. 2011 Jan 12;12:16. doi: 10.1186/1471-2105-12-16 (PMC3033325; doi:10.1186/1471-2105-12-16)
Supplement: Additional file 2 — Linkage disequilibrium plot for a simplified haplotype block structure. The figure included into this additional file describes a standard representation of pairwise dependences between genetic markers. [file 1471-2105-12-16-S2.PDF]

Linkage disequilibrium plot for a simplified haplotype block structure.

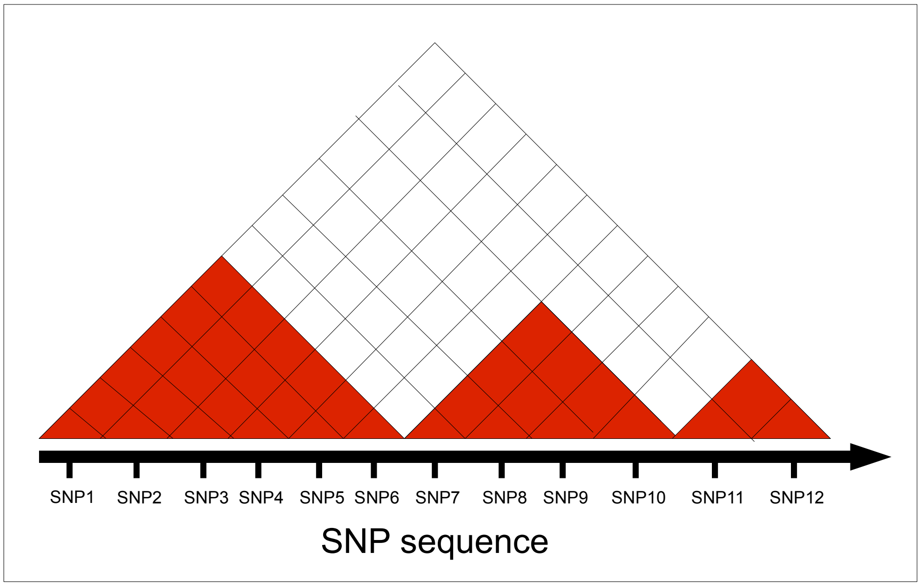

LD plot for a simplified haplotype block structure. LD (linkage disequilibrium) is revealed through the matrix of pairwise dependences between genetic markers. For a pair of SNPs, the color shade is all the darker as the dependence between the two SNPs is high.
