# Supplementary material for: A hierarchical Bayesian network approach for linkage disequilibrium modeling and data-dimensionality reduction prior to genome-wide association studies
Source: BMC Bioinformatics. 2011 Jan 12;12:16. doi: 10.1186/1471-2105-12-16 (PMC3033325; doi:10.1186/1471-2105-12-16)
Supplement: Additional file 3 — Linkage disequilibrium plot of a real 500 kb SNP sequence. The figure presented in this additional file shows the linkage disequilibrium plot of a real 500 kb SNP sequence. [file 1471-2105-12-16-S3.PDF]

Linkage disequilibrium plot of a real 500 kb SNP sequence.

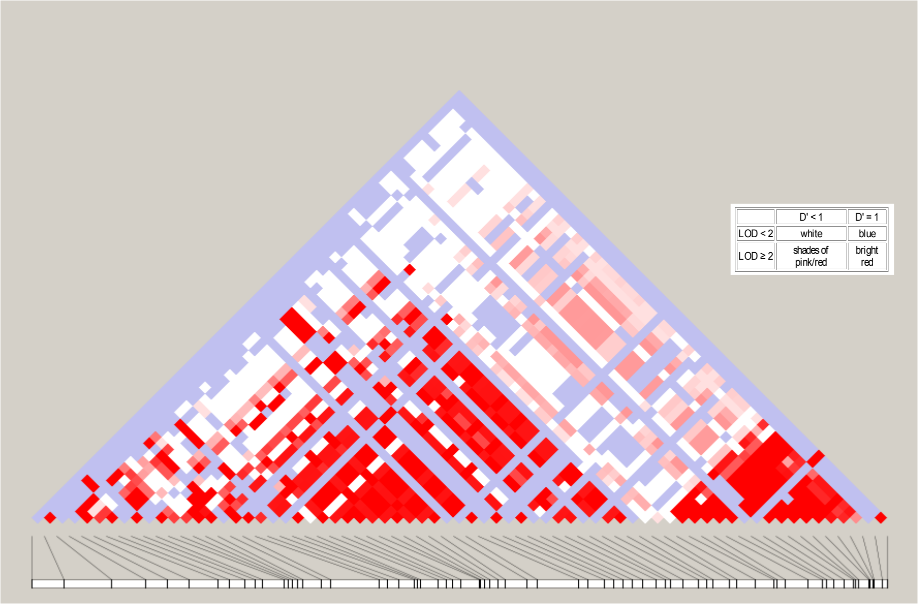

LD plot of a real 500 kb SNP sequence. Human genome, chromosome 1, region [10 000 kb - 10 500 kb]. LD (linkage disequilibrium) is revealed through the matrix of pairwise dependences between genetic markers. For a pair of SNPs, the color shade is all the darker as the dependence between the two SNPs is high.
