# Supplementary material for: A hierarchical Bayesian network approach for linkage disequilibrium modeling and data-dimensionality reduction prior to genome-wide association studies
Source: BMC Bioinformatics. 2011 Jan 12;12:16. doi: 10.1186/1471-2105-12-16 (PMC3033325; doi:10.1186/1471-2105-12-16)
Supplement: Additional file 4 — Hierarchical latent class model. The figure presented in this additional file depicts a hierarchical latent class model. [file 1471-2105-12-16-S4.PDF]

Hierarchical latent class model.

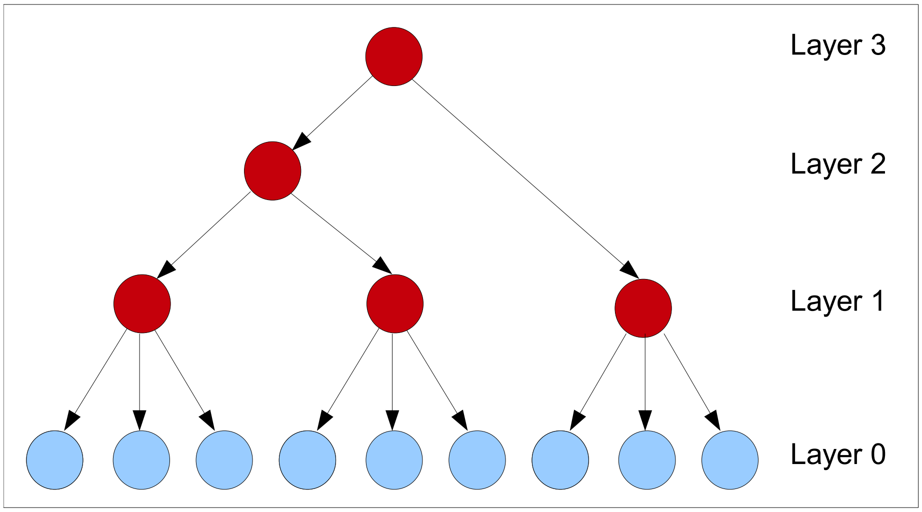

Hierarchical latent class model. The light shade indicates the observed variables whereas the dark shade points out the latent variables.
