# Supplementary material for: A hierarchical Bayesian network approach for linkage disequilibrium modeling and data-dimensionality reduction prior to genome-wide association studies
Source: BMC Bioinformatics. 2011 Jan 12;12:16. doi: 10.1186/1471-2105-12-16 (PMC3033325; doi:10.1186/1471-2105-12-16)
Supplement: Additional file 6 — Parameter value adjustment for the generation of simulated genotypic data through software HAPSIMU. The table included in this additional file enumerates the values chosen for the parameters of software HAPSIMU. [file 1471-2105-12-16-S6.PDF]

Parameter value adjustment for the generation of simulated genotypic data through software HAPSIMU.

disease model parameters	disease prevalence	0.01
	genotype relative risk	1.5
	frequency of disease susceptible allele	min: 0.1, max: 0.3
population structure model parameters	proportion of YRI* in cases	0.47
	proportion of YRI in controls	0.53
	number of generations	5
	frequency difference	min: 0, max: 0.06
simulation parameters	sample size (number of individuals)	**2000**
	proportion of cases in total sample	0.5
	simulating times	1
	genotype missing rate	0

*in reference to the African population included in the HapMap project (Yoruba in Ibadan, Nigeria, see http://hapmap.ncbi.nlm.nih.gov/).
