# Supplementary material for: A hierarchical Bayesian network approach for linkage disequilibrium modeling and data-dimensionality reduction prior to genome-wide association studies
Source: BMC Bioinformatics. 2011 Jan 12;12:16. doi: 10.1186/1471-2105-12-16 (PMC3033325; doi:10.1186/1471-2105-12-16)
Supplement: Additional file 7 — Average running time versus number of variables. The figure presented in this additional file plots the running time of the CFHLC algorithm versus the number of SNPs in the dataset. [file 1471-2105-12-16-S7.PDF]

Average running time versus number of variables.

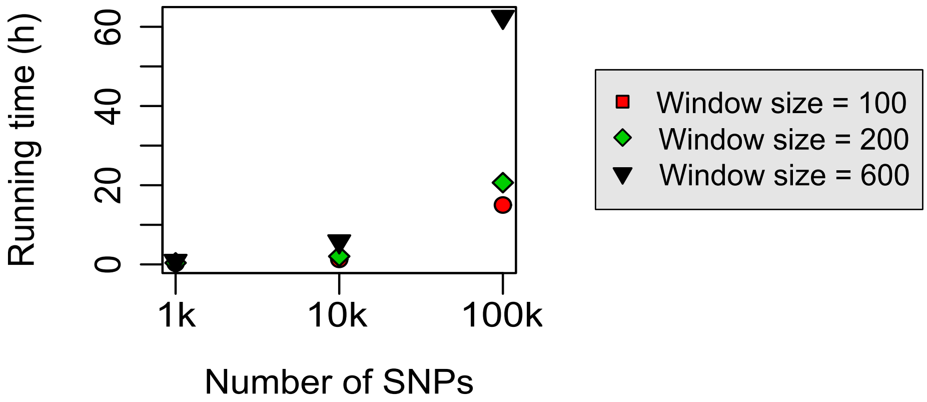

Average running time versus number of variables. Twenty benchmarks were considered. $s = 100$, $a = 0.2$, $b = 2, card_{max} = 20$, $t_{CAST} = 0.95$, $t_{MI} = quantile_{MI}(0.5)$, $t = 0.5$ (for CFHLC parameter description, see Text, Section Algorithm).
