# Supplementary material for: A hierarchical Bayesian network approach for linkage disequilibrium modeling and data-dimensionality reduction prior to genome-wide association studies
Source: BMC Bioinformatics. 2011 Jan 12;12:16. doi: 10.1186/1471-2105-12-16 (PMC3033325; doi:10.1186/1471-2105-12-16)
Supplement: Additional file 8 — Impact of window size on running time. The figure presented in this additional file plots the running time of the CFHLC algorithm versus the window size. [file 1471-2105-12-16-S8.PDF]

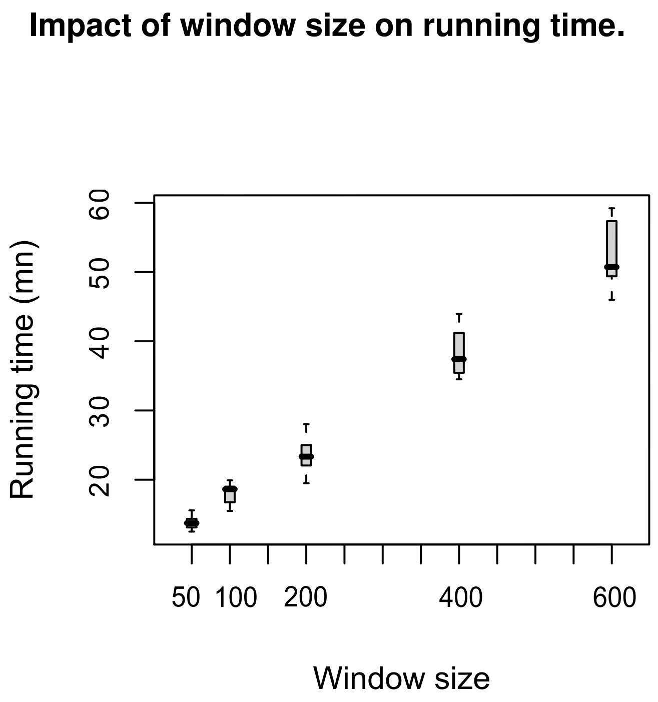

Impact of window size on running time. Average on 20 benchmarks. 1000 SNPs processed, $a = 0.2$, $b = 2$, $card_{max} = 20$, $t_{CAST} = 0.95$, $t_{MI} = quantile_{MI}(0.5)$, $t = 0.5$ (for CFHLC parameter description, see Text Section Algorithm).

This additional file more thoroughly describes the influence of the window size increase on running time. Interestingly, we observe an unexpected linear tendency of the running time. Actually, two phenomena act in opposite directions. The first one increases the number of LCMs to be learnt: the larger the window size is, the more layers there are in the FHLCM. The second phenomenon entails a decrease in the number of LCMs to be learnt: with CAST parameters maintained constant (mutual information threshold t_{MI} and similarity threshold t_{CAST}), the larger the total number of variables to be partitioned, the larger the clusters. Larger clusters are expected to decrease the number of LCMs in the FHLCM.
