# Supplementary material for: A hierarchical Bayesian network approach for linkage disequilibrium modeling and data-dimensionality reduction prior to genome-wide association studies
Source: BMC Bioinformatics. 2011 Jan 12;12:16. doi: 10.1186/1471-2105-12-16 (PMC3033325; doi:10.1186/1471-2105-12-16)
Supplement: Additional file 9 — Number of variables per layer over the whole FHLC model. The figure included in this additional file describes the average distribution of the variables over the layers (over 20 benchmarks). [file 1471-2105-12-16-S9.PDF]

Number of variables per layer over the whole FHLC model.

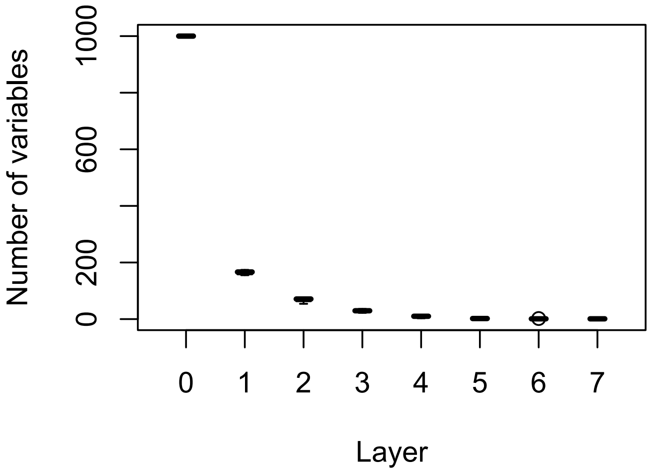

Number of variables per layer over the whole FHLC model. The distribution has been estimated over 20 benchmarks. 1000 SNPs processed, $s = 100$, $a = 0.2$, $b = 2$, $card_{max} = 20$, $t_{CAST} = 0.95$, $t_{MI} = quantile_{MI}(0.5)$, $t = 0.5$ (for CFHLC parameter description, see text, Section Algorithm).

We observe a dramatical decrease of the number of variables per layer (over the whole FHLCM) as the layer increases. 64% of the latent variables are present in the first layer. In particular, the decrease between the first and second layers amounts to 60%.
