# Supplementary material for: A hierarchical Bayesian network approach for linkage disequilibrium modeling and data-dimensionality reduction prior to genome-wide association studies
Source: BMC Bioinformatics. 2011 Jan 12;12:16. doi: 10.1186/1471-2105-12-16 (PMC3033325; doi:10.1186/1471-2105-12-16)
Supplement: Additional file 10 — Impact of window size on the number of roots. The figure included in this additional file depicts the impact of window size on the number of roots. [file 1471-2105-12-16-S10.PDF]

Impact of window size on the number of roots.

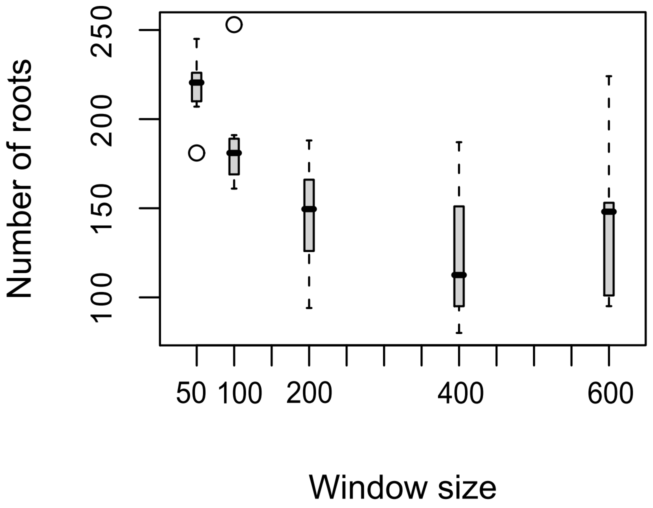

Impact of window size on the number of roots. Average on 20 benchmarks. 1000 SNPs processed, $a = 0.2$, $b = 2$, $card_{max} = 20$, $t_{CAST} = 0.95$, $t_{MI} = quantile_{MI}(0.5)$, $t = 0.5$ (for CFHLC parameter description, see text, Section Algorithm).

Interestingly, this additional file highlights the decrease in the number of variables to be tested for association with the disease (from 1000 observed variables to less than 200 forest roots in the case "$s = 100$"). In this case, algorithm CFHLC allows a reduction in the number of variables of more than 80%.
