# Supplementary material for: A hierarchical Bayesian network approach for linkage disequilibrium modeling and data-dimensionality reduction prior to genome-wide association studies
Source: BMC Bioinformatics. 2011 Jan 12;12:16. doi: 10.1186/1471-2105-12-16 (PMC3033325; doi:10.1186/1471-2105-12-16)
Supplement: Additional file 11 — Impact of window size on the number of latent variables. The figure presented in this additional file shows the impact of window size on the number of latent variables. [file 1471-2105-12-16-S11.PDF]

Impact of window size on the number of latent variables.

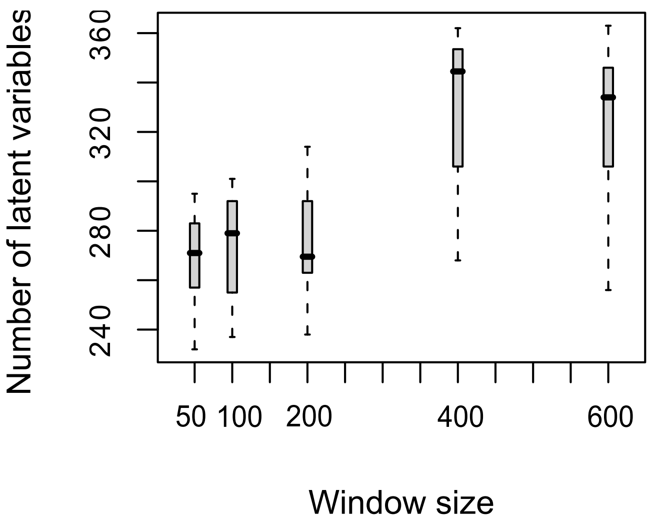

Impact of window size on the number of latent variables. Average on 20 benchmarks. 1000 SNPs processed, $a = 0.2$, $b = 2$, $card_{max} = 20$, $t_{CAST} = 0.95$, $t_{MI} = quantile_{MI}(0.5)$, $t = 0.5$ (for CFHLC parameter description, see text, Section Algorithm).

The number of latent variables increases with the window size. This increase with window size is due to the fact that more higher-order interactions are taken into account. In average, around 270 latent variables and 5 to 6 layers are reported for the case "$s = 100$", whereas around 340 latent variables and 8 layers are identified for the case "$s = 600$".
