# Supplementary material for: A hierarchical Bayesian network approach for linkage disequilibrium modeling and data-dimensionality reduction prior to genome-wide association studies
Source: BMC Bioinformatics. 2011 Jan 12;12:16. doi: 10.1186/1471-2105-12-16 (PMC3033325; doi:10.1186/1471-2105-12-16)
Supplement: Additional file 12 — Impact of window size on the number of layers. The figure presented in this additional file describes the impact of window size on the number of layers. [file 1471-2105-12-16-S12.PDF]

Impact of window size on the number of layers.

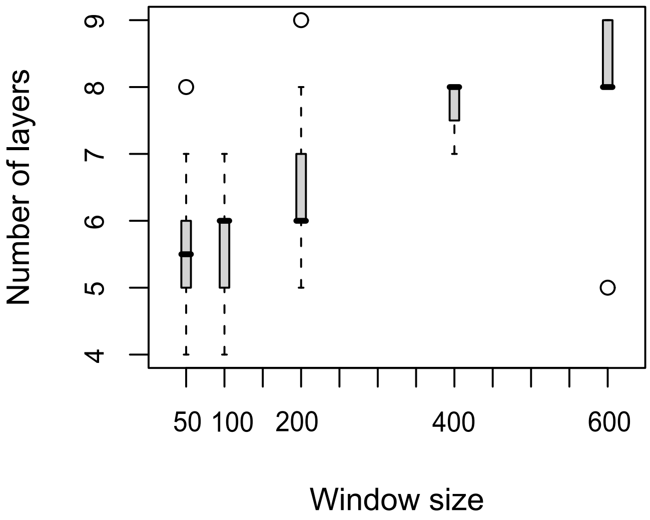

Impact of window size on the number of layers. Average on 20 benchmarks. 1000 SNPs processed, $a = 0.2$, $b = 2$, $card_{max} = 20$, $t_{CAST} = 0.95$, $t_{MI} = quantile_{MI}(0.5)$, $t = 0.5$ (for CFHLC parameter description, see text, Section Algorithm).

Like the number of latent variables, the number of layers increases with the window size.
