# Supplementary material for: A hierarchical Bayesian network approach for linkage disequilibrium modeling and data-dimensionality reduction prior to genome-wide association studies
Source: BMC Bioinformatics. 2011 Jan 12;12:16. doi: 10.1186/1471-2105-12-16 (PMC3033325; doi:10.1186/1471-2105-12-16)
Supplement: Additional file 13 — Impact of window size of the number of latent variables per layer and on the ratio of the number of latent variables per layer to the total number of variables. The two subfigures included in this additional file depict the impact of window size on the number of latent variables per layer on the one hand and the impact of window size on the number of latent variables per layer to the total number of variables, on the other hand. [file 1471-2105-12-16-S13.PDF]

Impact of window size of the number of latent variables per layer and on the ratio of the number of latent variables per layer to the total number of variables.

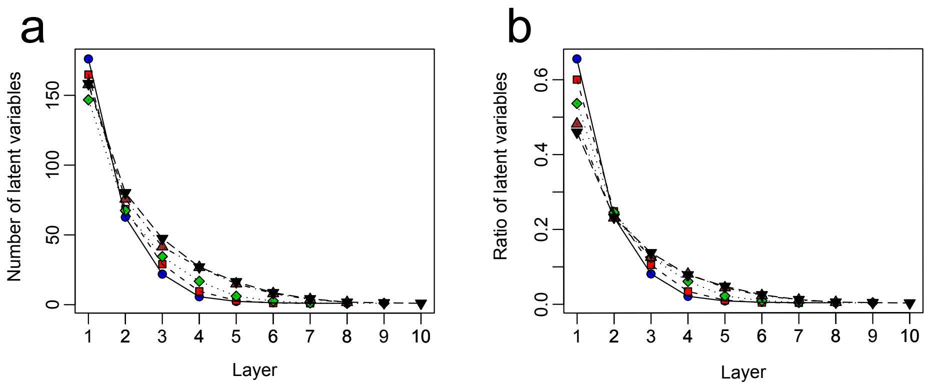

(a) Average number of latent variables per layer over the whole FHLC model; impact of window size. (b) Average ratio of the number of latent variables per layer to the total number of latent variables; impact of window size. Averages are computed over 20 benchmarks. 1000 SNPs processed, $s = 100$, $a = 0.2$, $b = 2$, $card_{max} = 20$, $t_{CAST} = 0.95$, $t_{MI} = quantile_{MI}(0.5)$, $t = 0.5$ (for CFHLC parameter description, see text, Section Algorithm).

This additional file provides a more thorough insight of the distributions of latent variables between layers. Figure (a) shows the impact of window size on the number of latent variables in a given layer while Figure (b) plots the ratios of the number of latent variables per layer to the total number of latent variables. Again, we observe a constant result: for all layers except the first one, the numbers (and ratios) are all the higher as the window size is larger. The exception relative to the first layer is explained as follows: when the number of observed variables increases, that is when the window size increases, for a constant parameter setting of the partitioning algorithm, the number of clusters identified is smaller (with larger sizes).
