# Supplementary material for: A hierarchical Bayesian network approach for linkage disequilibrium modeling and data-dimensionality reduction prior to genome-wide association studies
Source: BMC Bioinformatics. 2011 Jan 12;12:16. doi: 10.1186/1471-2105-12-16 (PMC3033325; doi:10.1186/1471-2105-12-16)
Supplement: Additional file 14 — Impact of window size on scaled mutual information, per layer. The figure presented in this additional file describes the impact of window size on scaled mutual information, per layer, over the whole FHLC model. [file 1471-2105-12-16-S14.PDF]

## Impact of window size on scaled mutual information per layer over the whole FHLC model.

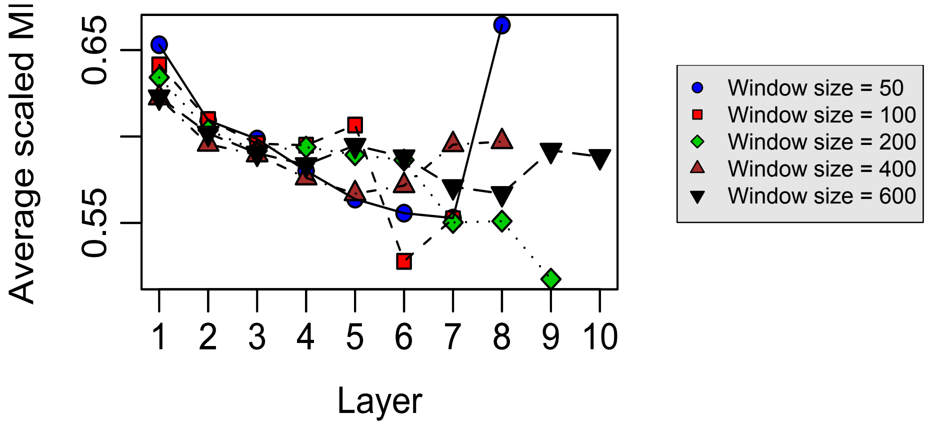


**Average scaled mutual information per layer over the whole FHLC model; impact of window size.** Average on 20 benchmarks. 1000 SNPs processed, $a = 0.2$, $b = 2$, $card_{max} = 20$, $t_{CAST} = 0.95$, $t_{MI} = quantile_{MI}(0.5)$, $t = 0.5$ (for CFHLC parameter description, see text, Section Algorithm).

This additional file displays how information fades while the layer number increases. Regarding window size 600, the first four layers show average values around 0.62, 0.60, 0.59 and 0.58 for the scaled mutual information-based score $C$. In the highest layers, average scaled mutual information is at least equal to 0.52 and 0.56 for the cases "$s = 100$" and "$s = 600$" respectively. Therefore, not only is a major point reached regarding tractability, information dilution is also controlled in an efficient way. The increasing variance is explained by the decreasing number of latent variables involved in the average calculation. In the highest layers, this sampling effect is all the more acute, entailing such variations as that observed for layer 8 and window size 50. In the latter case, a unique latent variable has been considered to compute the mean; it happened that the mutual information-based score calculated was outstandingly high in comparison with other values.
