# Supplementary material for: A hierarchical Bayesian network approach for linkage disequilibrium modeling and data-dimensionality reduction prior to genome-wide association studies
Source: BMC Bioinformatics. 2011 Jan 12;12:16. doi: 10.1186/1471-2105-12-16 (PMC3033325; doi:10.1186/1471-2105-12-16)
Supplement: Additional file 15 — Average scaled mutual information per layer over the whole FHLC model; impact of parameters a and b. The figure presented in this additional file shows the impact of parameters a and b on scaled mutual information, per layer, over the whole FHLC model. [file 1471-2105-12-16-S15.PDF]

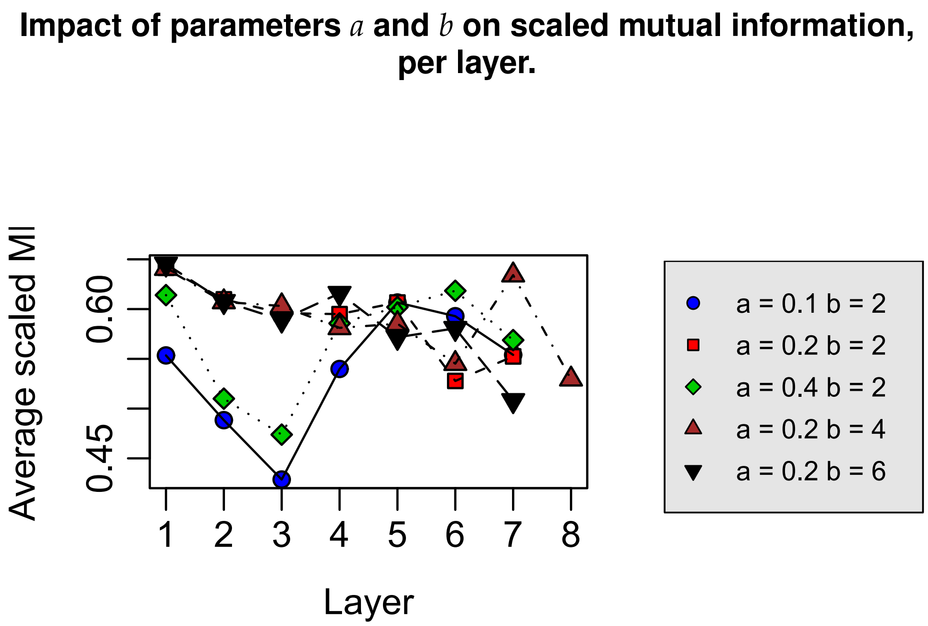

Average scaled mutual information per layer over the whole FHLC model; impact of parameters *a* and *b*. Average over 20 benchmarks. 1000 SNPs processed, $s = 100$, $card_{max} = 20$, $t_{CAST} = 0.95$, $t_{MI} = quantile_{MI}(0.5)$, $t = 0.5$ (for CFHLC parameter description, see text, Section Algorithm).

As expected, average scaled mutual information raises with parameters *a* and *b* because latent variables with larger cardinalities allow to capture more information about their child nodes, in the FHLCM.
